# Analysis of normal and osteoarthritic canine cartilage mRNA expression by quantitative polymerase chain reaction

**DOI:** 10.1186/ar2053

**Published:** 2006-10-10

**Authors:** Dylan N Clements, Stuart D Carter, John F Innes, William ER Ollier, Philip JR Day

**Affiliations:** 1 The Musculoskeletal Research Group, c/o Department of Veterinary Pathology, Faculty of Veterinary Science, University of Liverpool, Liverpool, L69 3BX, UK; 2 Centre for Integrated Genomic Medical Research, The Stopford Building, University of Manchester, Oxford Road, Manchester, M13 9PT, UK

## Abstract

The molecular basis to mammalian osteoarthritis (OA) is unknown. We hypothesised that the expression of selected proteases, matrix molecules, and collagens believed to have a role in the pathogenesis of OA would be changed in naturally occurring canine OA cartilage when compared to normal articular cartilage. Quantitative (real-time) reverse transcriptase-polymerase chain reaction assays were designed measuring the expression of selected matrix molecules (collagens and small leucine-rich proteoglycans), key mediators of the proteolytic degradation of articular cartilage (metalloproteinases, cathepsins), and their inhibitors (tissue inhibitors of matrix metalloproteinases). All data were normalised using a geometric mean of three housekeeping genes, and the results subjected to power calculations and corrections for multiple hypothesis testing. We detected increases in the expression of *BGN*, *COL1A2*, *COL2A1*, *COL3A1*, *COL5A1*, *CSPG2*, *CTSB*, *CTSD*, *LUM*, *MMP13*, *TIMP1*, and *TNC *in naturally occurring canine OA. The expression of *TIMP2 *and *TIMP4 *was significantly reduced in canine OA cartilage. The patterns of gene expression change observed in naturally occurring canine OA were similar to those reported in naturally occurring human OA and experimental canine OA. We conclude that the expression profiles of matrix-associated molecules in end-stage mammalian OA may be comparable but that the precise aetiologies of OA affecting specific joints in different species are presently unknown.

## Introduction

Osteoarthritis (OA) is the most common debilitating disease of mammalian joints. The clinical prevalence of human OA has been estimated to affect 12.1% of the population aged 25 to 74 [1], whereas clinical OA affects up to 20% of the canine population at large [2]. Canine OA usually develops secondary to an identifiable initiating cause (for example, secondary to hip dysplasia [3]), although it can be experimentally induced [4]. Experimental models provide controlled and reproducible development of OA [5], but only the study of naturally occurring disease allows experimental findings to be directly related to the clinical presentation with absolute certainty. The relatedness of the pathogenesis of a common disease, such as OA, in two different species has not been characterised [6].

At present, the precise mechanisms underlying the molecular pathogenesis of OA are unknown. Quantification of gene expression is a fundamental tool for investigating gene function in biological systems, particularly for elucidating pathological mechanisms at play in diseased tissues. Quantitative reverse transcriptase-polymerase chain reaction (RT-PCR) is currently considered the most accurate technique for quantifying gene expression. With the publication of the canine genome [7], RT-PCR assays can now be readily designed for the measurement of canine gene expression. Although canine-specific oligonucleotide microarrays are available for the quantification of mRNA transcripts in canine tissue, such as cartilage [8], quantitative RT-PCR validation of the results produced is still required.

Articular cartilage is composed of chondrocytes embedded in an extracellular matrix (ECM). The structural strength of the matrix is provided by collagens such as type II collagen (*COL2*), type VI collagen (*COL6*), type IX collagen (*COL9*), type XI collagen (*COL11*), and type XVI collagen (*COL16*), with *COL2 *accounting for 90% to 95% of the collagen composition of the ECM. Other than water, the major non-collagenous component of articular cartilage is aggrecan (*AGC1*); smaller components include the small leucine-rich proteoglycans such as biglycan (*BGN*), chondroitin sulphate proteoglycan 2 (*CSPG2*), decorin (*DCN*), lumican (*LUM*), and tenascin C (*TNC*). The proteolytic degradation of normal and osteoarthritic cartilage matrix is performed by proteases such as the matrix metalloproteinases (*MMPs*) [9], members of the *ADAMTS *(a disintegrin and metalloproteinase with thrombospondin-like motif) family (or 'aggrecanases') [10], and lysosomal proteases (such as cathepsins) [11]. Tissue inhibitors of matrix metalloproteinases (*TIMP*s) are naturally occurring inhibitors of *MMP *and *ADAMTS *function [12]. The authors are unaware of any publications documenting the change in expression of structural ECM and protease collagens in the articular cartilage of dogs with naturally occurring OA. We hypothesised that the expression of selected proteases, matrix molecules, and collagens would be modulated in naturally occurring canine OA.

## Materials and methods

### Cartilage samples

Osteoarthritic articular cartilage was harvested from the femoral heads of dogs that had end-stage naturally occurring OA secondary to hip dysplasia (*n *= 15, mean age 2.7 years [range 1 to 12 years], mean weight 28.2 kg [range 25 to 36 kg]) and that were undergoing routine surgical treatment of the disease (total hip replacement). In all cases, severe clinical and radiographic signs associated with OA of the affected joint necessitated surgical treatment of the disease. Articular cartilage was harvested from the area surrounding the central cartilage erosion usually observed on the canine OA hip [3]. Normal articular cartilage was harvested without visual evidence of hip dysplasia or OA from the femoral heads of dogs, which had been euthanatised for reasons unrelated to joint disease (*n *= 13, mean age 3.3 years [range 1 to 11 years], mean weight 26.2 kg [range 15 to 40 kg]). Articular cartilage was obtained from the same site of the femoral head in the control dogs as it was in diseased dogs. Cartilage samples were immediately immersed in RNAlater™ (Ambion Ltd., Huntingdon, UK) at room temperature for 24 hours before being stored at -20°C until use, in accordance with the manufacturer's instructions.

### RNA extraction from articular cartilage

Tissue samples were removed from RNAlater™ and total RNA was extracted using phenol/guanidine HCl reagents (Trizol™; Invitrogen Ltd, Paisley, UK) and isolated as previously described [13,14]. An on-column DNA digestion step was included (RNase-Free DNase Set; Qiagen Ltd, Crawley, UK). Final elution of the total RNA was performed using 30 μl of RNase-free water and repeated to maximise the amount of RNA eluted.

### RNA quality assessment

The concentration of total RNA of each sample was quantified by using a spectrophotometer (NanoDrop Technologies, Wilmington, DE, USA). RNA integrity was analysed by evaluating the capillary electrophoresis trace (Agilent 2100 Bioanalyser; Agilent Technologies, Santa Clara, CA, USA) of the sample by using the RNA integrity number (RIN) algorithm [15], degradation factor (DF) [16], and ribosomal peak ratio. The sample was determined to have minimal or no loss of integrity (RIN >6.4 and/or DF <10 and/or a ribosomal ratio >0.4) and thus deemed suitable for use in the following experiments in accordance with a previously developed quality algorithm [14].

### Synthesis of cDNA

Each sample was normalised to a concentration of 20 μg/μl, using RNAse-free water, and reverse transcription was performed using 10 μl RNA (200 μg total RNA) with oligo-dT_12–18 _and Superscript II reverse transcriptase (Invitrogen Ltd). After reverse transcription, the template was diluted with 500 μl RNase/DNase-free water. cDNA was stored at -80°C until later use in quantitative PCR.

### Quantitative PCR

Transcript sequences were obtained from the canine genome database [17], with cross-reference to the National Center for Biotechnology Information (Bethesda, MD, USA) [18]. Where possible, assays were designed in areas of sequence showing 100% homology between predicted and verified sequences. Primer and probe sequences were designed using online design software [19]. To enhance the probability of transcript-specific PCR, selected amplicon systems were designed so that the last six to seven bases of a 3' primer or the probe crossed an exon-exon boundary. When this was not possible, the primers were designed to be hybridised on different exons, with an intronic sequence greater than 1,100 base pairs, to maintain specificity for mRNA. Some assays could be designed within only a single exon, and thus a genomic DNA assay was also designed to determine whether genomic contamination was present. BLAST (Basic Local Alignment Search Tool) searches were performed for all primer sequences to confirm gene specificity.

Genes were selected for assay on the basis of their importance to cartilage homeostasis or pathology as derived from a literature review of naturally occurring human OA and experimental canine OA and from the results of a preliminary canine-specific whole genome microarray study, using a small number of samples. Assays were designed for quantification of expression of five collagen genes (type I collagen, alpha 2 chain [*COL1A2*], type II collagen alpha 1 chain [*COL2A1*], type III collagen alpha 1 chain [*COL3A1*], type V collagen alpha 1 chain [*COL5A1*], and type IX collagen alpha 3 chain [*COL9A3*]), six ECM genes (*AGC1*, *BGN*, *CSPG2*, *DCN*, *LUM*, and *TNC*), an intermediate filament (vimentin), proteases and their inhibitors (*ADAMTS-5*, cathepsins B [*CTSB*], cathepsin D [*CTSD*], *MMP13*, *TIMP1*, *TIMP2*, and *TIMP4*), and genomic DNA. Assays for four reference genes (glyceraldehyde-3-phosphate dehydrogenase [*GAPDH*], TATA box binding protein [*TBP*], ribosomal protein L13a [*RPL13A*], and succinate dehydrogenase complex, subunit A [*SDHA*]) (Table 1) were also designed. The reference genes used were selected from a panel of reference genes by applying a gene stability algorithm [20]. Primers were synthesised by MWG Biotech (London, UK). Locked nucleic acid fluorescence resonance energy transfer probes with a 5' reporter dye FAM (6-carboxy fluorescein) anda dark quencher dyewere synthesised by Roche Diagnostics Ltd (Lewes, West Sussex, UK).

The quantitative (real-time) PCR assays were all performed in triplicate using a TaqMan™ ABI PRISM 7900 SDS (Applied Biosystems, Foster City, CA, USA) in 384-well plate format. Each assay well had a 10-μl reaction volume consisting of 5 μl 2X PCR master mix with Uracil N-Glycosylase (Universal PCR Mastermix; Applied Biosystems), 0.1 μl each of 20 μM forward and reverse primers, 0.1 μl of 10 μM probe (Exiqon; Roche Diagnostics Ltd), and 4.7 μl of sample cDNA (templates) or water (negative controls).

The amplification was performed according to standard protocol with 10 minutes at 50°C followed by 40 cycles of 95°C for 1 minute and 60°C for 15 seconds, as recommended by the manufacturer (Applied Biosystems). Real-time data were analysed by using the Sequence Detection Systems software, version 2.2.1 (Applied Biosystems). The detection threshold was set manually at 0.05 for all assays. Standard curves were generated for each assay (Additional file 1), to confirm that all assays were generated within acceptable limits (efficiency 93% > x > 107.4%) and R^2 ^values (R^2 ^> 0.98) (with the exception of the genomic contamination assay, in which efficiency was lower, but the detection of any transcript was deemed unacceptable).

### Data analysis

The weights and ages of the patients were normally distributed and thus compared with the calculation of means and Student *t *tests. The weight of the articular cartilage samples and quantity of RNA extract were compared using median values and Mann-Whitney *U *tests because the data were not normally distributed.

Real-time data were analysed by generation of mean threshold cycle (C_T_) values from each transcript in triplicate. Geometric means [20] were calculated for the combined three reference genes (*GAPDH*, *TBP*, and *RPL13A*) and used to calculate the ΔΔC_T _(delta-delta C_T_) values and the relative amount of each target gene [21] (Table 2). A fourth reference gene (*SDHA*) was not included as a reference gene, because it was found to have differential expression between normal and OA samples, even when included as part of the normalisation calculation. The upper detection limit of dynamic range generated from the standard curves was used as a cut-off point, above which real-time data were discarded (that is, included in the statistical analyses as zero/no transcript present).

Data were compared with the calculations of means, standard deviations, and fold changes from normal and paired two-tailed *t *tests (body weight and age) performed in a spreadsheet program (Microsoft Excel 2003; Microsoft Corporation, Redmond, WA, USA) and the calculation of graphs, 95% confidence intervals (CIs) of the mean, and Mann-Whitney *U *tests (to compare the amount of each target) performed in a statistical analysis software package (Minitab version 14.1; Minitab Ltd., Coventry, UK). One-sided power calculations were performed, assuming normality from the two samples with unequal variance and using a freely available web-based program [22]. Significance was established at *p *< 0.05, and a robust statistical analysis was assumed to have a power value greater than or equal to 80%. Data were checked for errors due to multiple hypothesis testing by using the Benjamini and Hochberg false discovery rate (FDR) [23].

## Results

There were no significant differences between the ages (mean control 3.3 years [± 3.2 years, range 1 to 12 years], mean OA 2.7 years [± 3.1 years, range 1 to 11 years], *p *= 0.768) or body weights (mean control 26.2 kg [± 8.0 kg, range 15 to 32 kg], mean OA 28.3 kg [± 3.8 kg, range 23 to 36 kg], *p *= 0.109) of the dogs in the diseased and control groups. There was no significant difference between the weight of the cartilage samples (median control 103 mg [range 45 to 260 mg], median OA 92 mg [range 40 to 192 mg], *p *= 0.817) or the quantity of RNA extracted, as determined by spectrophotometer (median control 35 ng/μl [range 26 to 339 ng/μl], median OA 42 ng/μl [range 22 to 247 ng/μl], *p *= 0.788).

Expression values are presented in Table 3. Two genes were determined to have significant downregulation (*TIMP2 *and *TIMP4*) in canine OA cartilage. One gene was determined to be significantly downregulated (*SDHA*) but with a low power value (72%); this gene was excluded after FDR correction. Ten genes were determined to be significantly upregulated in the OA samples (*BGN*, *COL3A1*, *COL5A1*, *CSPG2*, *CTSB*, *CSTD*, *LUM*, *MMP13*, *TIMP1*, and *TNC*). Furthermore, in OA, three genes were determined to be upregulated (*COL1A2*, *COL2A1*, and *COL9A3*) but with low power values (74%, 78%, and 63%, respectively) and one gene was excluded after FDR correction (*COL9A3*).

**Figure 1:**
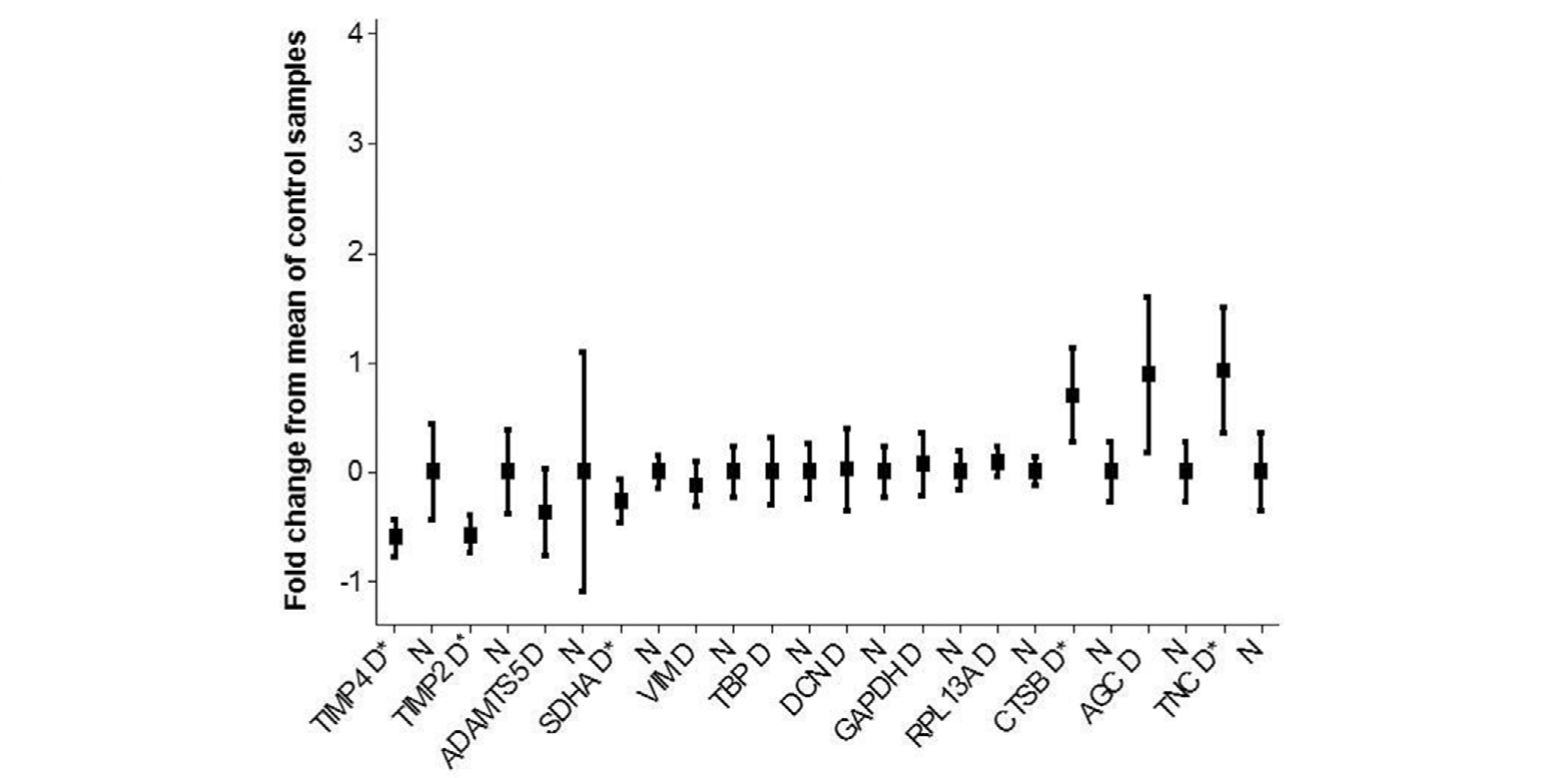
Graph illustrating the means and 95% confidence intervals (CIs) of the gene expression profiles. To normalise values, the mean of each control group has been used to normalise and produce fold changes in expression. The results of the *COL9A3 *transcript are omitted because the 95% CIs were very high. *Significant difference. *ADAMTS5*, ADAM metallopeptidase with thrombospondin type 1 motif, 5; *AGC*, aggrecan; *COL9A3*, type IX collagen alpha 3 chain; *CTSB*, cathepsin B; D, disease; *DCN*, decorin; *GAPDH*, glyceraldehyde-3-phosphate dehydrogenase; N, normal; *RPL13A*, ribosomal protein L13a; *SDHA*, succinate dehydrogenase complex, subunit A; *TBP*, TATA box binding protein; *TIMP2*, tissue inhibitor of metalloproteinase 2; *TIMP4*, tissue inhibitor of metalloproteinase 4; *TNC*, Tenascin C; *VIM*, vimentin.

**Figure 2:**
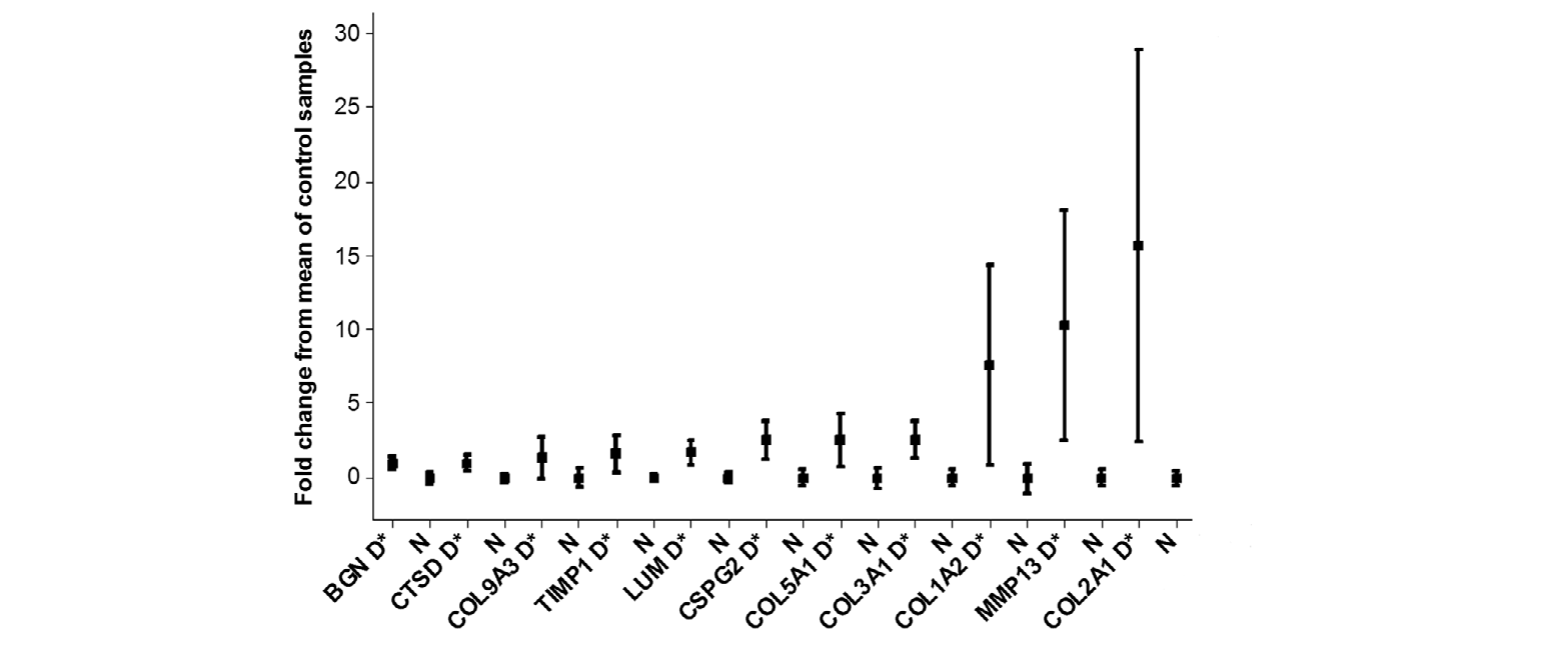
Graph illustrating the means and 95% confidence intervals (CIs) of the gene expression profiles. To normalise values, the mean of each control group has been used to normalise and produce fold changes in expression. The results of the *COL9A3 *transcript are omitted because the 95% CIs were very high. *Significant difference. *BGN*, biglycan; *COL1A2*, type I collagen, alpha two chain; *COL2A1*, type II collagen alpha 1 chain; *COL3A1*, type III collagen alpha 1 chain; *COL5A1*, type V collagen alpha 1 chain; *COL9A3*, type IX collagen alpha 3 chain; *CSPG2*, chondroitin sulphate proteoglycan 2; *CTSD*, cathepsin D; D, disease; *LUM*, lumican; *MMP13*, matrix metalloproteinase 13; N, normal; *TIMP1*, tissue inhibitor of metalloproteinase 1.

No amplification of genomic DNA was observed for any of the samples. The average standard deviation for the triplicates in each assay was 16.9% (range 7.3% to 37.9%), indicating that all assays were reproducible. Eleven of the 2,592 data points were removed because they were assumed to be aberrant (markedly different from the other two values in the triplicate). None of the 'no template' control wells (*n *= 864) revealed a signal. Fold gene expression changes are illustrated in Figures 1 and 2, with all data normalised to the mean of the control values (with a fold change of 0 being no change, a fold change of 1 meaning a doubling of expression, and a fold change of -0.5 meaning a halving of expression). Statistical and power calculations are reported in Table 3.

## Discussion

Quantitative (real-time) RT-PCR is the most sensitive technique for the determination of mRNA transcript number [24]. To maximise the precision of our data, we included only mRNA samples that had been determined as being of high quality (using an algorithm determined by previous work [15]), because mRNA degradation can affect assay performance [24]. Assays were optimised within specific limits of efficiency, and the dynamic range of each assay was determined, used, and presented with the expression data. Additionally, we corrected our results for multiple hypothesis testing (reducing the opportunity for making a statistical type II error) and present power values, allowing an interpretation of the strength of each significant up- or downregulation.

If variables such as the methods of mRNA extraction, RNA quality assessment, reverse transcription, assay design, measurement of genomic contamination, standard curve data generation, reference gene selection, and data normalisation were presented in the 'Materials and methods' and 'Results' sections of manuscripts using quantitative PCR, more appropriate comparison of results between different studies could be made. The geometric mean of three reference (housekeeping) genes was used in this study to reduce the variability associated with the use of a single reference gene. Geometric mean methodology has been validated as a more accurate normalisation technique than that using a single reference gene if the reference genes are selected through the use of a stability algorithm [21], although in this study one of the genes identified by the algorithm (*SDHA*) was not stably expressed (Table 3).

Gene expression varies with both the site of cartilage harvest [25] and the degree of cartilage degeneration [26] in the OA joint. We attempted to minimise this variability by using end-stage OA, age- and weight-matched samples, and stringent RNA quality control. A relatively high degree of heterogeneity (large 95% CIs) was observed in the level of gene expression measured from the clinical samples in this study, even existing between samples within the same group. This may reflect differences in dog age and/or breeds or variation in the time from surgical removal to collection in the preservative fluid. The analysis of additional samples or the phenotyping and selection of samples through histological grading may have increased the statistical powers of each of these differences observed, as the severity of OA measured by histology (Mankin score) correlates with a reduction in the expression of *COL2 *and *AGC *[27].

Cell culture-based biological systems provide a more controlled methodology for evaluating gene expression when compared with *in vivo *tissue. For example, increased cell numbers can be obtained, breed and age factors can be eradicated, and the absence of ECM facilitates the extraction of higher quality of mRNA [16]. This is particularly true for studies of smaller mammals such as the dog, in which clinical samples of osteoarthritic cartilage may be less than 100 mg in size. However, cell-based models may differ in both gene expression profiles [28] or cell phenotype [29] with *in vitro *tissue. Ultimately, our understanding of the molecular pathogenesis of OA requires relating changes observed with *in vitro *experimentation to those identified from clinical tissue.

The paucity of literature reporting changes in gene expression observed in naturally occurring canine OA implies that often this is not easy to achieve. In part, this reflects the difficulties associated with the use of clinical tissue samples, as noted above, and the fact that the technology required to enable the economic evaluation of gene expression across large groups of tissue samples is only just becoming available. Indeed, we were limited by sample quantity, quality, and cost and needed to rationalise our list of genes selected for evaluation, as discussed previously.

We document marked elevation of expression in genes encoding for collagen synthesis in the articular cartilage of dogs with end-stage OA, which concurs with the findings in early experimental canine OA [30-33]. *COL1A2*, *COL3A1*, and *COL5A1 *are characteristically synthesised by cells with a fibrocartilaginous phenotype [34].

The increased expression of *BGN*, *CSPG2*, *CTSB*, *DCN*, *LUM*, *MMP13*, and *TNC *is consistent with previous studies of expression of these genes in both naturally occurring human [35-39] and experimental canine OA [30-32]. The biological significance of fold changes in gene expression between control and OA samples is unknown in the absence of additional data such as gross, radiographic, or histological scoring or protein quantification. Likewise, the changes in gene expression documented do not specify whether these changes are causal or simply associated with the development of pathology in the OA joint.

We documented decreases in the expression of *TIMP2 *and *TIMP4 *and an increase in the expression of *TIMP1 *in canine OA cartilage. The decrease in *TIMP4 *expression was consistent with expression profiles of human OA cartilage [39], although *TIMP1 *expression has been documented as being decreased and *TIMP2 *expression has been documented as being unchanged in human OA [39]. Direct comparison of gene expression levels with those measured in other joints and/or in different species may be of limited value because the underlying aetiologies to the development of OA may differ. However, the evaluation of structural matrix components and proteases affecting those components is still of interest because the end-stage pathology characterising canine OA mimics that described for human OA [40].

## Conclusion

On the basis of the results we present, the gene expression of selected matrix molecules and key mediators of the proteolytic degradation of articular cartilage is changed in end-stage, naturally occurring OA of the canine hip. The patterns of gene expression change are broadly similar to those reported in experimental canine stifle OA and naturally occurring human OA.

## Abbreviations

*ADAMTS5 *= ADAM metallopeptidase with thrombospondin type 1 motif, 5; *AGC1 *= aggrecan; *BGN *= biglycan; CI = confidence interval; *COL1A2 *= type I collagen, alpha two chain; *COL2A1 *= type II collagen alpha 1 chain; *COL3A1 *= type III collagen alpha 1 chain; *COL5A1 *= type V collagen alpha 1 chain; *COL9A3 *= type IX collagen alpha 3 chain; *CSPG2 *= chondroitin sulphate proteoglycan 2; C_T _= mean threshold cycle; *CTSB *= cathepsin B; *CTSD *= cathepsin D; *DCN *= decorin; DF = degradation factor; ECM = extracellular matrix; FDR = false discovery rate; *GAPDH *= glyceraldehyde-3-phosphate dehydrogenase; LUM = lumican; *MMP *= matrix metalloproteinase; OA = osteoarthritis; RIN = RNA integrity number; *RPL13A *= ribosomal protein L13a; RT-PCR = reverse-transcriptase-polymerase chain reaction; *SDHA *= succinate dehydrogenase complex, subunit A; TBP = TATA box binding protein; *TIMP1 *= tissue inhibitor of metalloproteinase 1; *TIMP2 *= tissue inhibitor of metalloproteinase 2; *TIMP4 *= tissue inhibitor of metalloproteinase 4; *TNC *= tenascin C.

## Competing interests

The Universal Probe Library™ was supplied at a reduced cost by Roche Diagnostics Ltd.

## Authors' contributions

DNC collected and processed samples, carried out the molecular genetic studies, performed the statistical analysis, and drafted the manuscript. SDC, JFI, and WERO conceived of the study, participated in its design and coordination, and helped to draft the manuscript. PJRD participated in the design of the study, the assay design, and statistical analysis. All authors read and approved the final manuscript.

## Supplementary Material

Additional file 1 Standard curves generated for each assay by ten fold serial dilutions of template,withreal-time data analysed by using the Sequence Detection Systems software, version 2.2.1 (Applied Biosystems). Click here for file

## Figures and Tables

**Table 1:** A list of primer and probe sequences for the genes evaluated

Gene	Forward	Reverse	Probe
*ADAMTS5*	TGGGTTCCCAAATATGCAG	CTGTCCCATCCGTCACCT	CTGGGAGA
*1AGC1*	GGGACCTGTGTGAGATCGAC	GTAACAGTGGCCCTGGAACT	AGGAGCTG
*BGN*	CAGAACAACGACATCTCAGAGC	TCACCAGGACGAGAGCGTA	CTCCACCA
*COL1A2*	CTATCAATGGTGGTACCCAGTTT	TGTTTTGAGAGGCATGGTTG	GCCTGCTG
*COL2A1*	CTGGTGAACCTGGACGAGAG	ACCACGATCACCCTTGACTC	CCTCCTGG
*COL3A1*	GGATGGTGGCTTCCAGTTT	CCAGCTGGACATCGAGGA	GCTGCCTG
*COL5A1*	AACCTGTCGGATGGCAAGT	CAGTCCAAGATCAAGGTGACAT	CAGCATCC
*COL9A3*	CGAGGTGCCTCAGGTGAC	ACCCAGCTCTCCTTTGTCC	GAGACCAG
*CSPG2*	TGGATGGTTTTAATACGTTCAGG	GCCGTAGTCACACGTCTCTG	CTGCCTTC
*CTSB*	CGGCCTTCACCGTGTACT	GTGACGTGCTGGTACACTCC	CTTCCTGC
*CTSD*	GGTCCACATGGAGCAGGT	TATGAGGGAGGTGCCTGTGT	TGGGCAGC
*DCN*	CGCTGTCAGTGCCATCTC	GGGGGAAGATCTTTTGGTACTT	TCCAGTGT
*GAPDH*	CTGGGGCTCACTTGAAAGG	CAAACATGGGGGCATCAG	CTGCTCCT
*Genomic*	AACCCTCAAAGATGAGGTTTAGC	ACTCTGGGATCACGCATGT	CTGCCTTC
*LUM*	ACCTGGAAATTCTTTTAATGTATCATC	CGGTATGTTTTTAAGCTTATTGTAGGA	TGCTGGAG
*MMP13*	CCGCGACCTTATCTTCATCT	AACCTTCCAGAATGTCATAACCA	AGAGGCAG
*RPL13A*	CTGCCCCACAAGACCAAG	GGGATCCCATCAAACACCT	CCAGGCTG
*SDHA*	GGTGGCACTTCTACGACACC	ATGTAGTGGATGGCGTCCTG	CTGGCTGG
*TBP*	TCCACAGCCTATCCAGAACA	CTGCTGCTGTTGTCTCTGCT	CTGGAGGA
*TIMP1*	TGCATCCTGCTGTTGCTG	AACTTGGCCCTGATGACG	CCCAGCAG
*TIMP2*	ATGGGCTGTGAGTGCAAGAT	CACTCATCCGGAGACGAGAT	CTGCCCCA
*TIMP4*	GCAGAGAGAAAGTCTGAATCATCA	GGCACTGTATAGCAGGTGGTAA	TGTGGCTG
*TNC*	TGGATGGGACAGTCAAGGA	GCTCAGCTCTGCCAGGTTA	CCACCTCC
*VIM*	TACAGGAAGCTGCTGGAAGG	CCTCAGGTTCAGGGAAGAAA	GAGCAGGA

*ADAMTS5*, ADAM metallopeptidase with thrombospondin type 1 motif, 5; *AGC1*, aggrecan; *BGN*, biglycan; *COL1A2*, type I collagen, alpha two chain; *COL2A1*, type II collagen alpha 1 chain; *COL3A1*, type III collagen alpha 1 chain; *COL5A1*, type V collagen alpha 1 chain; *COL9A3*, type IX collagen alpha 3 chain; *CSPG2*, chondroitin sulphate proteoglycan 2; *CTSB*, cathepsin B; *CTSD*, cathepsin D; *DCN*, decorin; *GAPDH*, glyceraldehyde-3-phosphate dehydrogenase; *Genomic*, Genomic DNA, *LUM*, lumican;*MMP13*, matrix metalloproteinase 13; *RPL13A*, ribosomal protein L13a; *SDHA*, succinate dehydrogenase complex, subunit A; *TBP*, TATA box binding protein; *TIMP1*, tissue inhibitor of metalloproteinase 1; *TIMP2*, tissue inhibitor of metalloproteinase 2; *TIMP4*, tissue inhibitor of metalloproteinase 4; *TNC*, Tenascin C; *VIM*, vimentin.

**Table 2:** The dynamic range, standard curve slope, R^2 ^value, and efficiency of each polymerase chain reaction assay

Assay	Lower detection limit (C_T _value)	Upper detection limit (C_T _value)	Standard curve slope	R^2 ^value	Efficiency
*ADAMTS5*	26.0	35.9	-3.32	0.99	100.2
*AGC*	18.5	34.7	-3.29	0.99	101.5
*BGN*	20.8	34.8	-3.49	1.00	93.3
*COL1A2*	17.4	33.5	-3.30	1.00	101.0
*COL2A1*	22.7	32.2	-3.22	1.00	104.6
*COL3A1*	16.5	33.0	-3.33	1.00	99.9
*COL5A1*	23.2	33.1	-3.31	1.00	100.5
*COL9A3*	26.3	32.7	-3.22	1.00	104.8
*CSPG2*	21.4	34.3	-3.25	1.00	103.2
*CTSB*	19.7	32.6	-3.24	1.00	103.3
*CTSD*	24.1	34.2	-3.29	1.00	101.5
*DCN*	19.0	31.9	-3.25	1.00	103.0
*GAPDH*	22.7	35.2	-3.27	0.99	102.3
*Genomic*	16.8	40.0	-4.42	1.00	68.3
*LUM*	19.9	33.7	-3.48	1.00	93.9
*MMP13*	26.1	36.3	-3.36	0.98	98.6
*RPL13A*	18.6	32.1	-3.36	1.00	98.6
*SDHA*	21.6	34.6	-3.26	1.00	102.5
*TBP*	16.5	30.0	-3.39	1.00	97.4
*TIMP1*	22.6	33.1	-3.48	1.00	93.7
*TIMP2*	21.8	32.1	-3.43	1.00	95.7
*TIMP4*	29.5	35.8	-3.16	0.99	107.4
*TNC*	20.1	33.0	-3.26	1.00	102.5
*VIM*	15.8	32.7	-3.35	1.00	98.8

See Additional file 1 for further details. *ADAMTS5*, ADAM metallopeptidase with thrombospondin type 1 motif, 5; *AGC1*, aggrecan; *BGN*, biglycan; *COL1A2*, type I collagen, alpha two chain; *COL2A1*, type II collagen alpha 1 chain; *COL3A1*, type III collagen alpha 1 chain; *COL5A1*, type V collagen alpha 1 chain; *COL9A3*, type IX collagen alpha 3 chain; *CSPG2*, chondroitin sulphate proteoglycan 2; *CTSB*, cathepsin B; *CTSD*, cathepsin D; *DCN*, decorin; *GAPDH*, glyceraldehyde-3-phosphate dehydrogenase; *LUM*, lumican;*MMP13*, matrix metalloproteinase 13; *RPL13A*, ribosomal protein L13a; *SDHA*, succinate dehydrogenase complex, subunit A; *TBP*, TATA box binding protein; *TIMP1*, tissue inhibitor of metalloproteinase 1; *TIMP2*, tissue inhibitor of metalloproteinase 2; *TIMP4*, tissue inhibitor of metalloproteinase 4; *TNC*, Tenascin C; *VIM*, vimentin.

**Table 3:** Change in gene expression, mean 2^-ΔΔCT ^values, significance and power of comparisons between normal and OA canine articular cartilage

Gene	Number of samples in which expression was detected	2^-ΔΔCT ^normal	2^-ΔΔCT ^OA	Fold change in expression (diseased versus normal)	Mann-Whitney *U *test *p *value	Power
*TIMP4*	27	0.109	0.043	-0.608	0.0094	0.859
*TIMP2*	28	3.959	1.664	-0.580	0.0020	0.844
*ADAMTS5*	16	0.031	0.019	-0.551	0.8478	0.175
*SDHA*	28	0.323	0.234	-0.275	0.0476	0.722
*VIM*	28	32.742	28.909	-0.117	0.5493	0.195
*TBP*	28	0.106	0.106	0.001	0.8178	0.051
*DCN*	28	73.034	74.253	0.017	0.5190	0.059
*GAPDH*	28	1.548	1.648	0.064	0.9633	0.105
*RPL13A*	28	7.048	7.722	0.096	0.3814	0.275
*CTSB*	26	0.280	0.476	0.698	0.0060	0.886
*AGC*	28	0.082	0.155	0.887	0.1670	0.778
*TNC*	28	2.700	5.205	0.927	0.0099	0.886
*BGN*	28	15.511	30.984	0.998	0.0043	0.976
*CTSD*	28	0.148	0.295	0.999	0.0066	0.944
*COL9A3*	27	0.231	0.546	1.365	0.0304	0.633
*TIMP1*	28	0.551	1.468	1.663	0.0008	0.853
*LUM*	28	1.635	4.476	1.738	0.0015	0.991
*CSPG2*	27	0.079	0.279	2.530	0.0005	0.981
*COL5A1*	28	0.615	2.188	2.555	0.0069	0.887
*COL3A1*	26	10.573	37.867	2.581	0.0011	0.982
*COL1A2*	28	0.805	6.941	7.621	0.0043	0.737
*MMP13*	26	0.014	0.161	10.322	0.0010	0.857
*COL2A1*	27	1.412	23.583	15.705	0.0001	0.779

*ADAMTS5*, ADAM metallopeptidase with thrombospondin type 1 motif, 5; *AGC*, aggrecan; *BGN*, biglycan; *COL1A2*, type I collagen, alpha two chain; *COL2A1*, type II collagen alpha 1 chain; *COL3A1*, type III collagen alpha 1 chain; *COL5A1*, type V collagen alpha 1 chain; *COL9A3*, type IX collagen alpha 3 chain; *CSPG2*, chondroitin sulphate proteoglycan 2; *CTSB*, cathepsin B; *CTSD*, cathepsin D; *DCN*, decorin; *GAPDH*, glyceraldehyde-3-phosphate dehydrogenase; *LUM*, lumican; *MMP13*, matrix metalloproteinase 13; OA, osteoarthritis; *RPL13A*, ribosomal protein L13a; *SDHA*, succinate dehydrogenase complex, subunit A; *TBP*, TATA box binding protein; *TIMP1*, tissue inhibitor of metalloproteinase 1; *TIMP2*, tissue inhibitor of metalloproteinase 2; *TIMP4*, tissue inhibitor of metalloproteinase 4; *TNC*, Tenascin C; *VIM*, vimentin.

## References

[B1] Lawrence RC, Helmick CG, Arnett FC, Deyo RA, Felson DT, Giannini EH, Heyse SP, Hirsch R, Hochberg MC, Hunder GG (1998). Estimates of the prevalence of arthritis and selected musculoskeletal disorders in the United States. Arthritis Rheum.

[B2] RIMADYL^® ^(carprofen) website. http://www.rimadyl.com.

[B3] Burton-Wurster N, Farese JP, Todhunter RJ, Lust G (1999). Site-specific variation in femoral head cartilage composition in dogs at high and low risk for development of osteoarthritis: insights into cartilage degeneration. Osteoarthritis Cartilage.

[B4] Pond MJ, Nuki G (1973). Experimentally-induced osteoarthritis in the dog. Ann Rheum Dis.

[B5] Brandt KD (2002). Animal models of osteoarthritis. Biorheology.

[B6] Singh K, Masuda K, An HS (2005). Animal models for human disc degeneration. Spine J.

[B7] Lindblad-Toh K, Wade CM, Mikkelsen TS, Karlsson EK, Jaffe DB, Kamal M, Clamp M, Chang JL, Kulbokas EJ, Zody MC (2005). Genome sequence, comparative analysis and haplotype structure of the domestic dog. Nature.

[B8] Burton-Wurster N, Mateescu RG, Todhunter RJ, Clements KM, Sun Q, Scarpino V, Lust G (2005). Genes in canine articular cartilage that respond to mechanical injury: gene expression studies with Affymetrix canine GeneChip. J Hered.

[B9] Murphy G, Knauper V, Atkinson S, Butler G, English W, Hutton M, Stracke J, Clark I (2002). Matrix metalloproteinases in arthritic disease. Arthritis Res.

[B10] Nagase H, Kashiwagi M (2003). Aggrecanases and cartilage matrix degradation. Arthritis Res Ther.

[B11] Blanco Garcia FJ (1999). Catabolic events in osteoarthritic cartilage. Osteoarthritis Cartilage.

[B12] Baker AH, Edwards DR, Murphy G (2002). Metalloproteinase inhibitors: biological actions and therapeutic opportunities. J Cell Sci.

[B13] Reno C, Marchuk L, Sciore P, Frank CB, Hart DA (1997). Rapid isolation of total RNA from small samples of hypocellular, dense connective tissues. Biotechniques.

[B14] Clements DN, Vaughan-Thomas A, Peansukmanee S, Carter SD, Innes JF, Ollier WER, Clegg PD (2006). Assessment of the use of RNA quality metrics for the screening of normal and pathological canine articular cartilage samples. Am J Vet Res.

[B15] Imbeaud S, Graudens E, Boulanger V, Barlet X, Zaborski P, Eveno E, Mueller O, Schroeder A, Auffray C (2005). Towards standardization of RNA quality assessment using user-independent classifiers of microcapillary electrophoresis traces. Nucleic Acids Res.

[B16] Auer H, Lyianarachchi S, Newsom D, Klisovic MI, Marcucci G, Kornacker K (2003). Chipping away at the chip bias: RNA degradation in microarray analysis. Nat Genet.

[B17] Ensembl website. http://www.ensembl.org.

[B18] National Center for Biotechnology Information website. http://www.ncbi.nlm.nih.gov.

[B19] RocheAppliedScience website. http://www.roche-applied-science.com.

[B20] Vandesompele J, De Preter K, Pattyn F, Poppe B, Van Roy N, De Paepe A, Speleman F (2002). Accurate normalization of real-time quantitative RT-PCR data by geometric averaging of multiple internal control genes. Genome Biol.

[B21] Livak KJ, Schmittgen TD (2001). Analysis of relative gene expression data using real-time quantitative PCR and the 2-[Delta][Delta]CT method. Methods.

[B22] One sided power calculator. http://calculators.stat.ucla.edu/powercalc/.

[B23] Benjamini Y, Hochberg Y (1995). Controlling the false discovery rate: a practical and powerful approach to multiple testing. J Roy Statist Soc Ser B.

[B24] Bustin SA, Nolan T (2004). Pitfalls of quantitative real-time reverse-transcription polymerase chain reaction. J Biomol Tech.

[B25] Dumond H, Presle N, Pottie P, Pacquelet S, Terlain B, Netter P, Gepstein A, Livne E, Jouzeau J-Y (2004). Site specific changes in gene expression and cartilage metabolism during early experimental osteoarthritis. Osteoarthritis Cartilage.

[B26] Yagi R, McBurney D, Laverty D, Weiner S, Horton WE (2005). Intrajoint comparisons of gene expression patterns in human osteoarthritis suggest a change in chondrocyte phenotype. J Orthop Res.

[B27] Eid K, Thornhill TS, Glowacki J (2006). Chondrocyte gene expression in osteoarthritis: correlation with disease severity. J Orthop Res.

[B28] Zien A, Aigner T, Zimmer R, Lengauer T (2001). Centralization: a new method for the normalization of gene expression data. Bioinformatics.

[B29] Diaz-Romero J, Gaillard JP, Grogan SP, Nesic D, Trub T, Mainil-Varlet P (2005). Immunophenotypic analysis of human articular chondrocytes: changes in surface markers associated with cell expansion in monolayer culture. J Cell Physiol.

[B30] Matyas JR, Ehlers PF, Huang D, Adams ME (1999). The early molecular natural history of experimental osteoarthritis. I. Progressive discoordinate expression of aggrecan and type II procollagen messenger RNA in the articular cartilage of adult animals. Arthritis Rheum.

[B31] Lorenz H, Wenz W, Ivancic M, Steck E, Richter W (2005). Early and stable upregulation of collagen type II, collagen type I and YKL40 expression levels in cartilage during early experimental osteoarthritis occurs independent of joint location and histological grading. Arthritis Res Ther.

[B32] Adams ME, Matyas JR, Huang D, Dourado GS (1995). Expression of proteoglycans and collagen in the hypertrophic phase of experimental osteoarthritis. J Rheumatol Suppl.

[B33] Altman RD, Kates J, Chun LE, Dean DD, Eyre D (1992). Preliminary observations of chondral abrasion in a canine model. Ann Rheum Dis.

[B34] Benjamin M, Ralphs JR (2004). Biology of fibrocartilage cells. Int Rev Cytol.

[B35] Nishida Y, Shinomura T, Iwata H, Miura T, Kimata K (1994). Abnormal occurrence of a large chondroitin sulfate proteoglycan, PG-M/versican in osteoarthritic cartilage. Osteoarthritis Cartilage.

[B36] Cs-Szabo G, Melching LI, Roughley PJ, Glant TT (1997). Changes in messenger RNA and protein levels of proteoglycans and link protein in human osteoarthritic cartilage samples. Arthritis Rheum.

[B37] Pfander D, Heinz N, Rothe P, Carl HD, Swoboda B (2004). Tenascin and aggrecan expression by articular chondrocytes is influenced by interleukin 1{beta}: a possible explanation for the changes in matrix synthesis during osteoarthritis. Ann Rheum Dis.

[B38] Zhang H, Liew CC, Marshall KW (2002). Microarray analysis reveals the involvement of beta-2 microglobulin (B2M) in human osteoarthritis. Osteoarthritis Cartilage.

[B39] Kevorkian L, Young DA, Darrah C, Donell ST, Shepstone L, Porter S, Brockbank SM, Edwards DR, Parker AE, Clark IM (2004). Expression profiling of metalloproteinases and their inhibitors in cartilage. Arthritis Rheum.

[B40] Brandt KD, Myers SL, Burr D, Albrecht M (1991). Osteoarthritic changes in canine articular cartilage, subchondral bone, and synovium fifty-four months after transection of the anterior cruciate ligament. Arthritis Rheum.

