# Supplementary material for: Analysis of normal and osteoarthritic canine cartilage mRNA expression by quantitative polymerase chain reaction
Source: Arthritis Res Ther. 2006 Oct 10;8(6):R158. doi: 10.1186/ar2053 (PMC1794499; doi:10.1186/ar2053)
Supplement: Additional file 1 — Standard curves generated for each assay by ten fold serial dilutions of template,withreal-time data analysed by using the Sequence Detection Systems software, version 2.2.1 (Applied Biosystems). [file ar2053-S1.PDF]

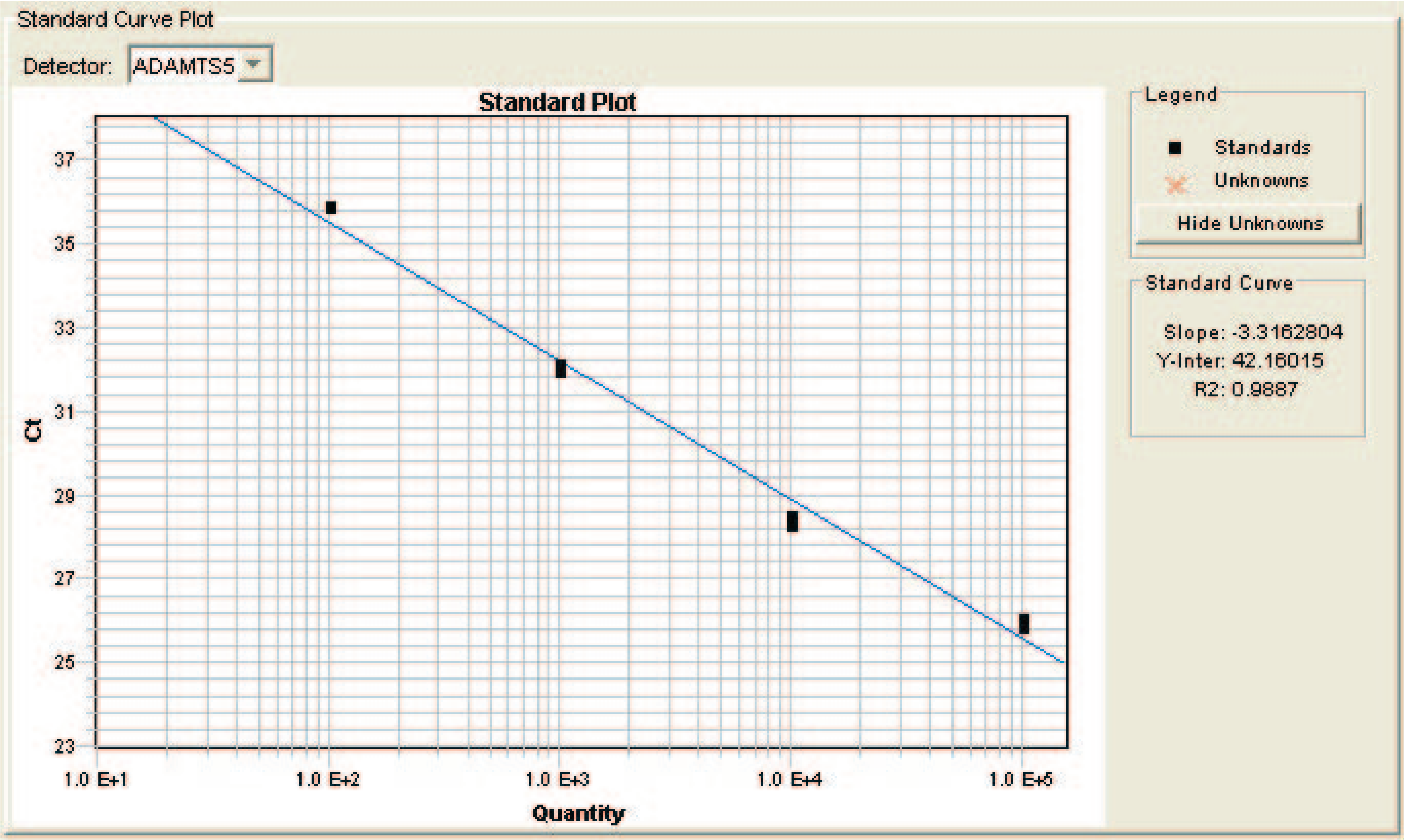
Standard Curve Plot
Detector: ADAMTS5
Standard Plot
Ct
37
35
33
31
29
27
25
23
1.0 E+1
1.0 E+2
1.0 E+3
1.0 E+4
1.0 E+5
Quantity
Legend
Standards
Unknowns
Hide Unknowns
Standard Curve
Slope: -3.3162804
Y-Inter: 42.16015
R2: 0.9887

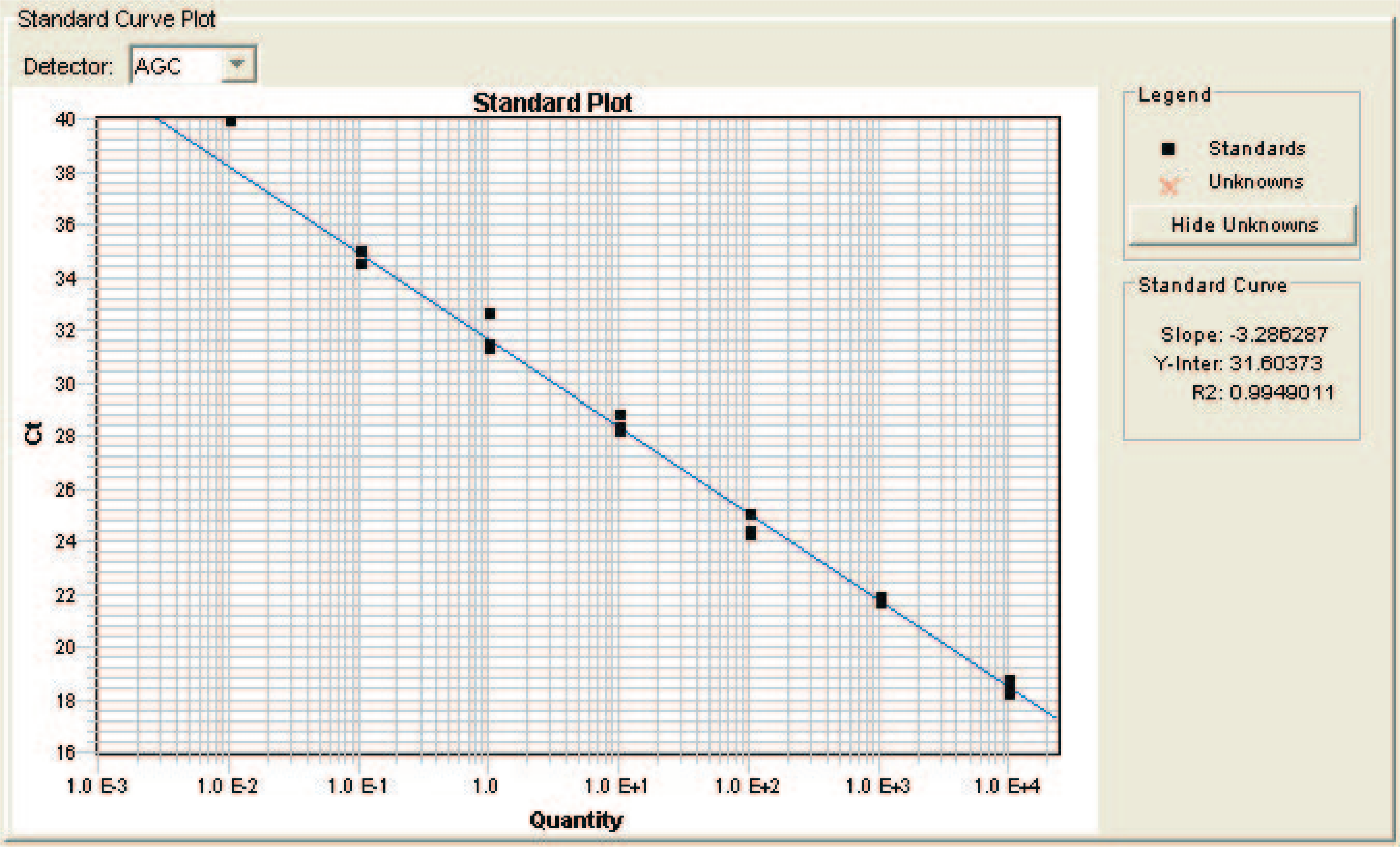
Standard Curve Plot
Detector: AGC
Standard Plot
Ct
40
38
36
34
32
30
28
26
24
22
20
18
16
1.0 E-3
1.0 E-2
1.0 E-1
1.0
1.0 E+1
1.0 E+2
1.0 E+3
1.0 E+4
Quantity
Legend
Standards
Unknowns
Hide Unknowns
Standard Curve
Slope: -3.286287
Y-Inter: 31.60373
R2: 0.9949011

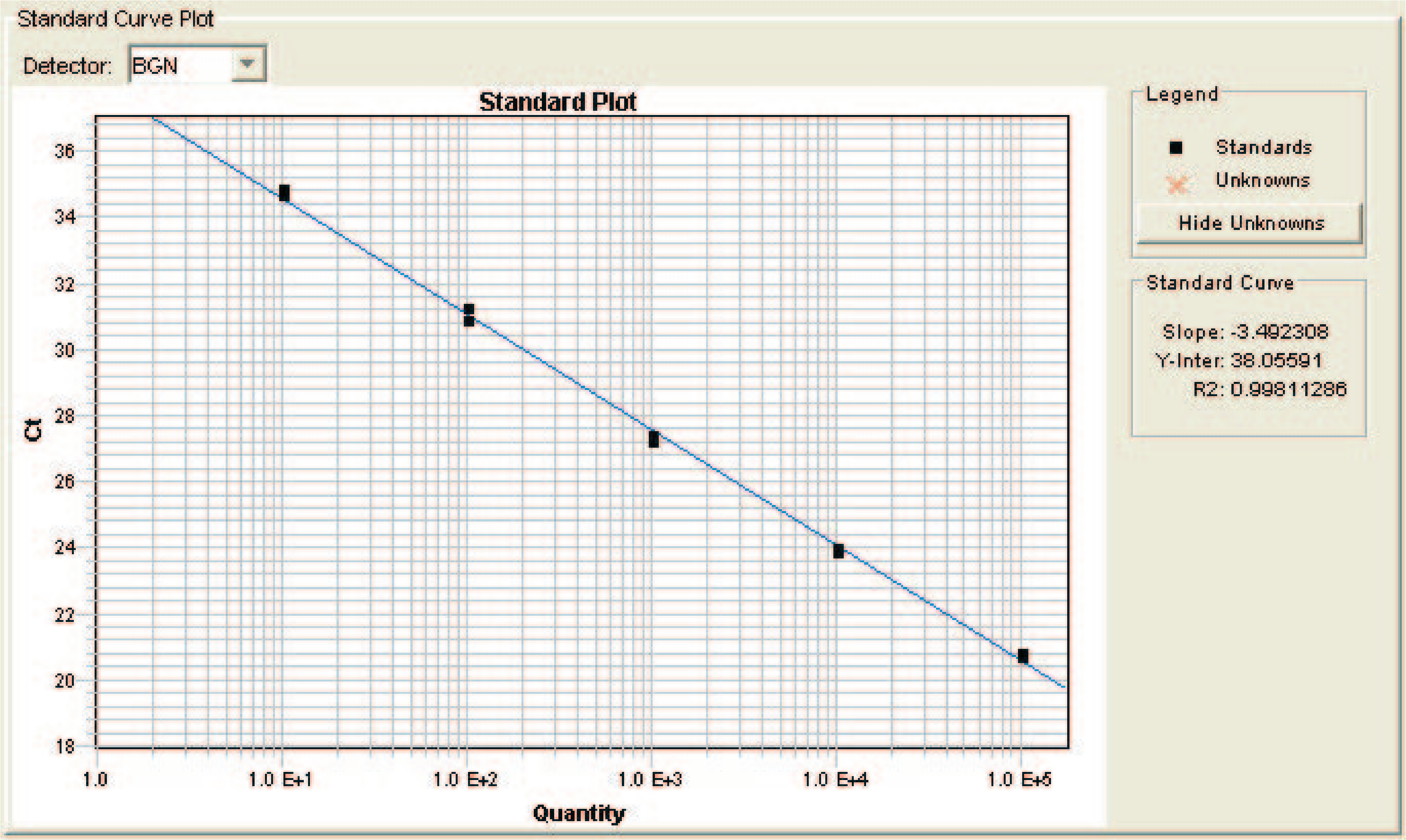

Standard Curve Plot
Detector: BGN
Standard Plot
Ct
36
34
32
30
28
26
24
22
20
18
1.0
1.0 E+1
1.0 E+2
1.0 E+3
1.0 E+4
1.0 E+5
Quantity
Legend
Standards
Unknowns
Hide Unknowns
Standard Curve
Slope: -3.492308
Y-Inter: 38.05591
R2: 0.99811286

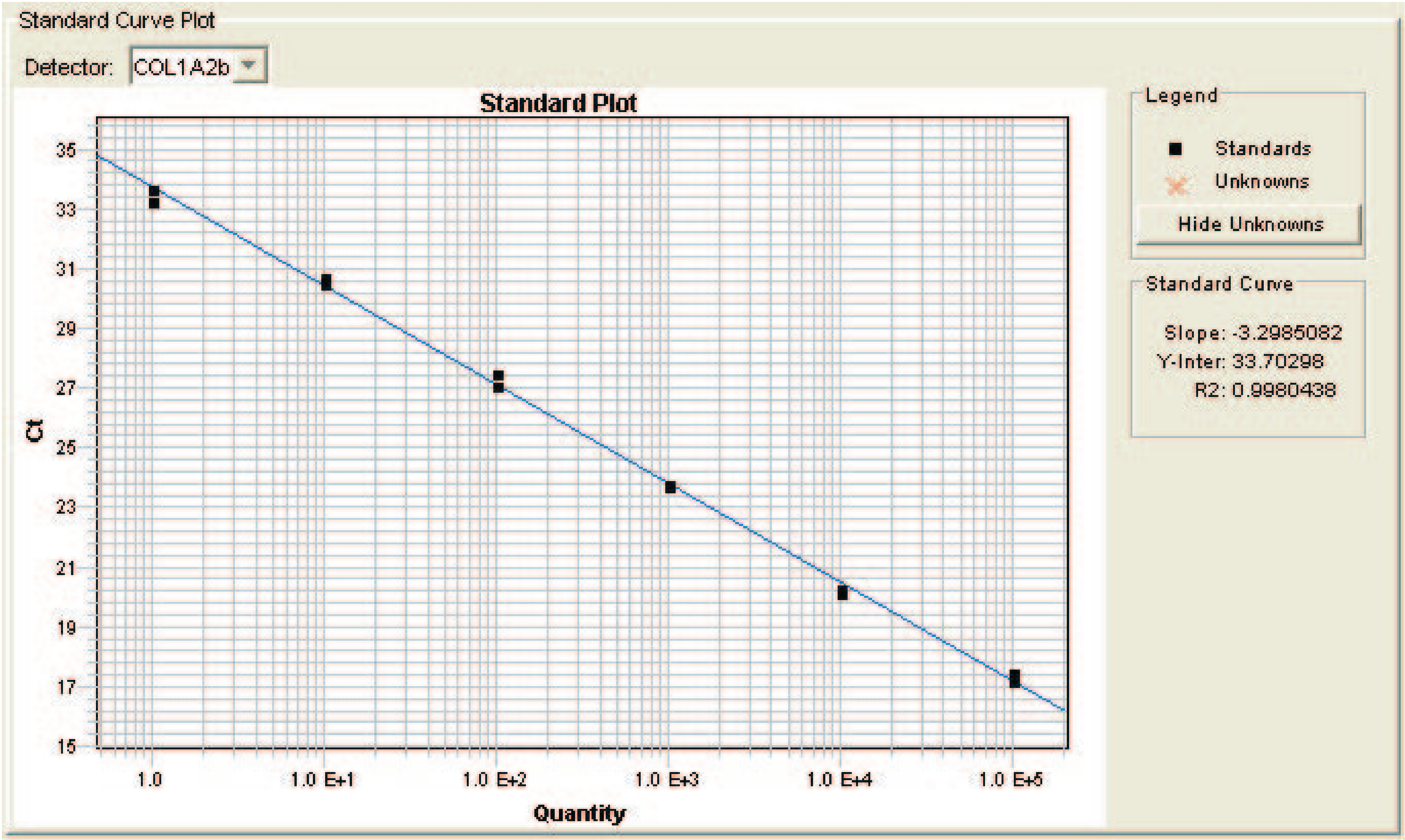
Standard Curve Plot
Detector: COL1A2b
Standard Plot
Ct
35
33
31
29
27
25
23
21
19
17
15
1.0
1.0 E+1
1.0 E+2
1.0 E+3
1.0 E+4
1.0 E+5
Quantity
Legend
Standards
Unknowns
Hide Unknowns
Standard Curve
Slope: -3.2985082
Y-Inter: 33.70298
R2: 0.9980438

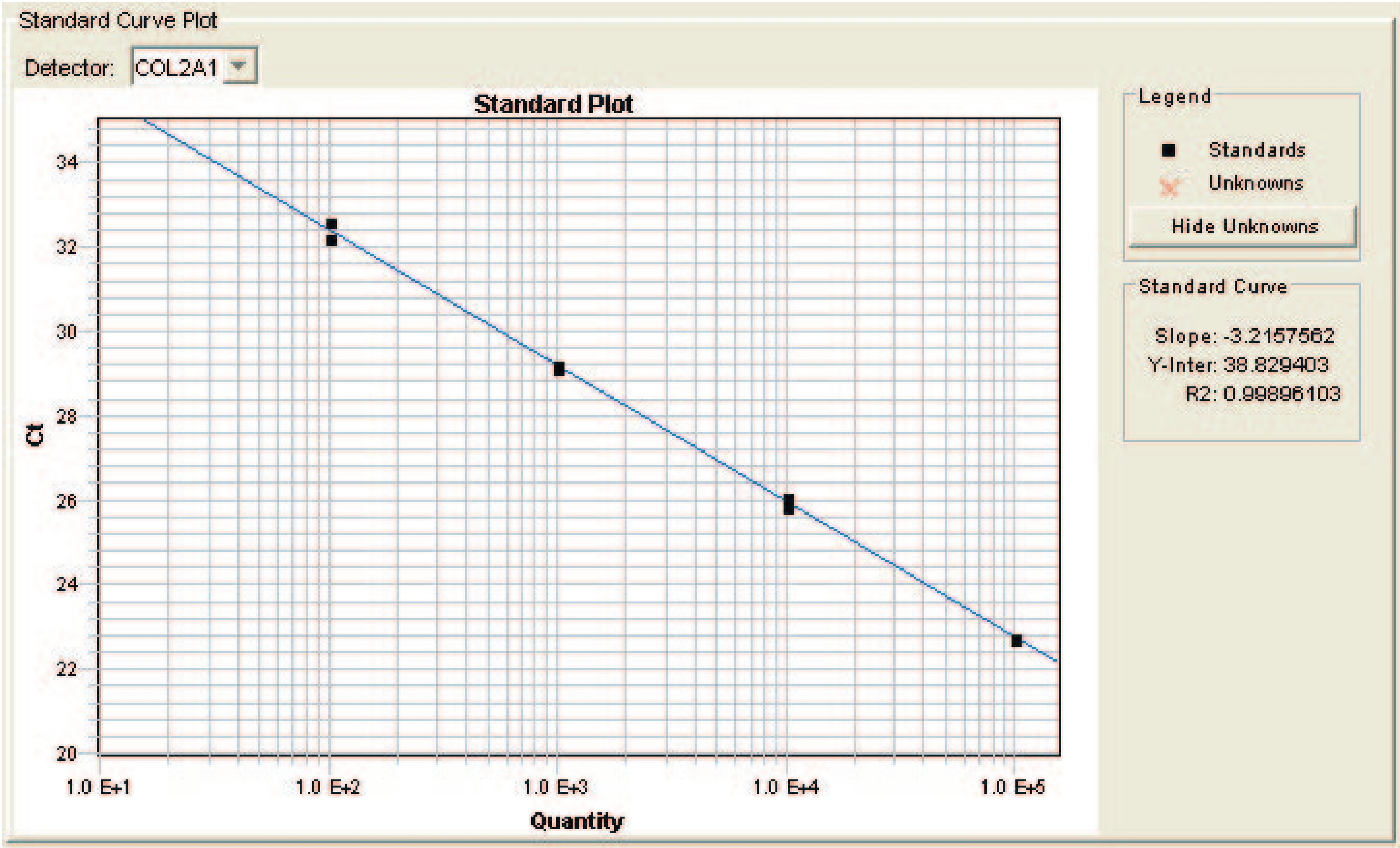
Standard Curve Plot
Detector: COL2A1
Standard Plot
Ct
34
32
30
28
26
24
22
20
1.0 E+1
1.0 E+2
1.0 E+3
1.0 E+4
1.0 E+5
Quantity
Legend
Standards
Unknowns
Hide Unknowns
Standard Curve
Slope: -3.2157562
Y-Inter: 38.829403
R2: 0.99896103

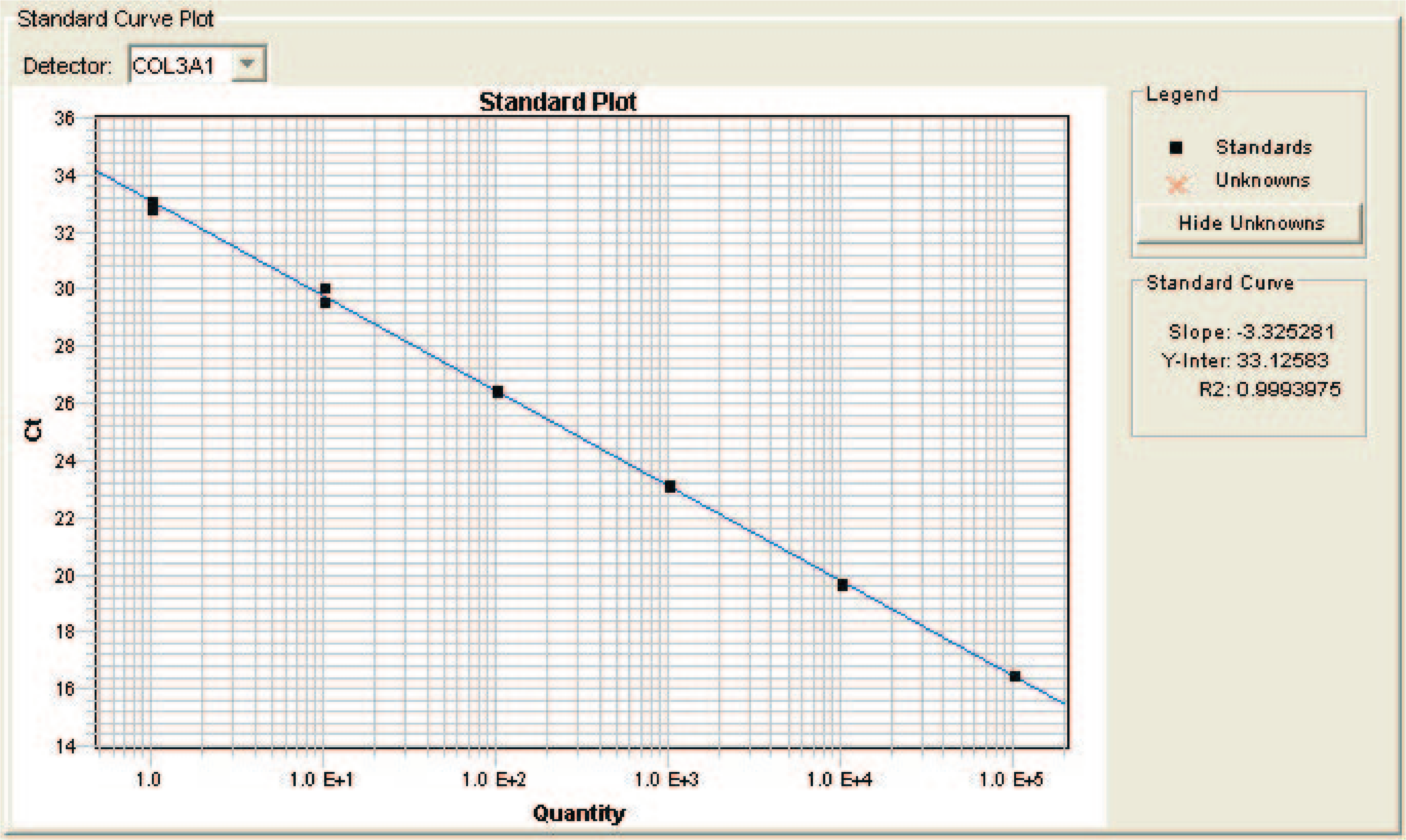

Standard Curve Plot
Detector: COL3A1
Standard Plot
Ct
36
34
32
30
28
26
24
22
20
18
16
14
1.0
1.0 E+1
1.0 E+2
1.0 E+3
1.0 E+4
1.0 E+5
Quantity
Legend
Standards
Unknowns
Hide Unknowns
Standard Curve
Slope: -3.325281
Y-Inter: 33.12583
R2: 0.9993975

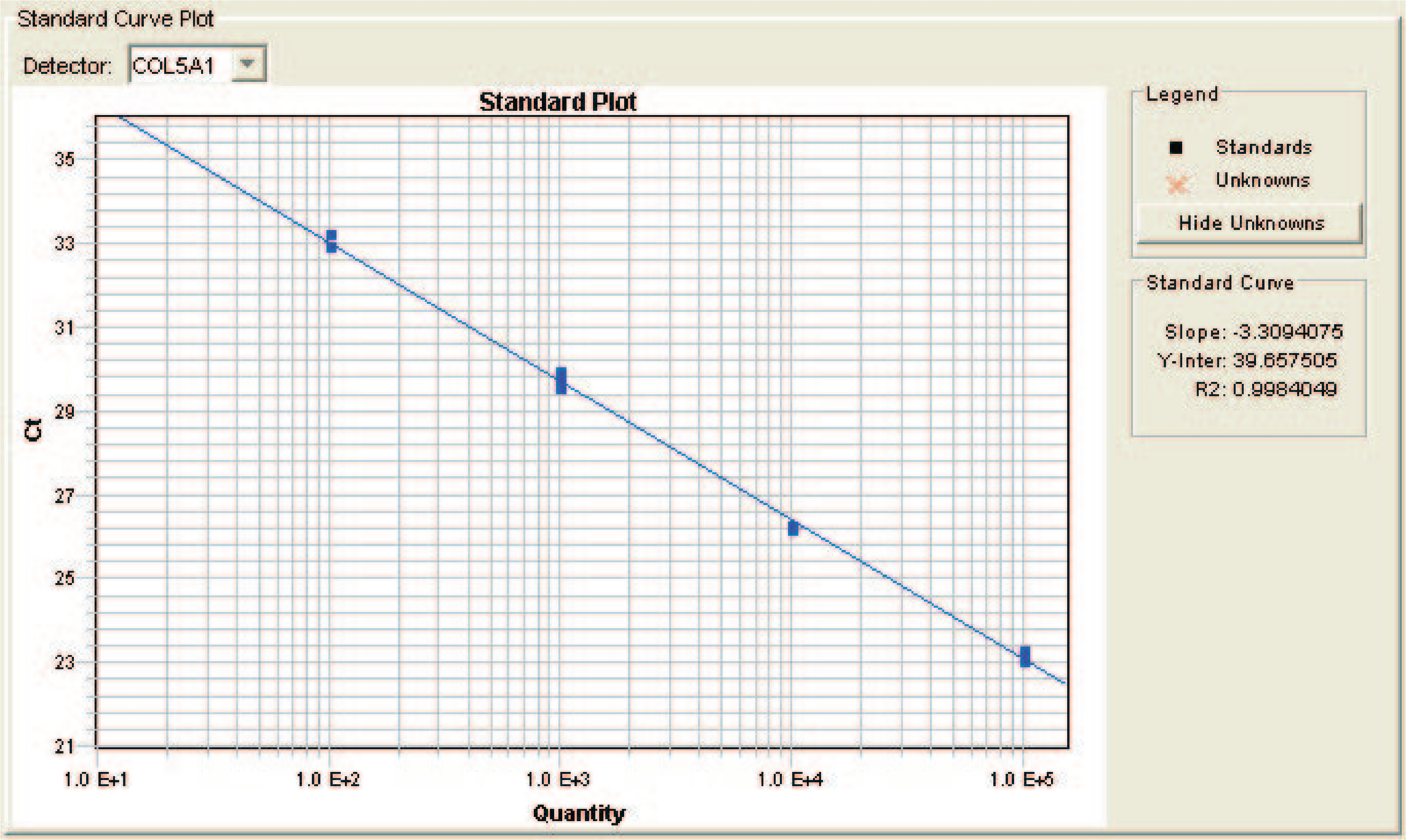
Standard Curve Plot
Detector: COL5A1
Standard Plot
Ct
35
33
31
29
27
25
23
21
1.0 E+1
1.0 E+2
1.0 E+3
1.0 E+4
1.0 E+5
Quantity
Legend
Standards
Unknowns
Hide Unknowns
Standard Curve
Slope: -3.3094075
Y-Inter: 39.657505
R2: 0.9984049

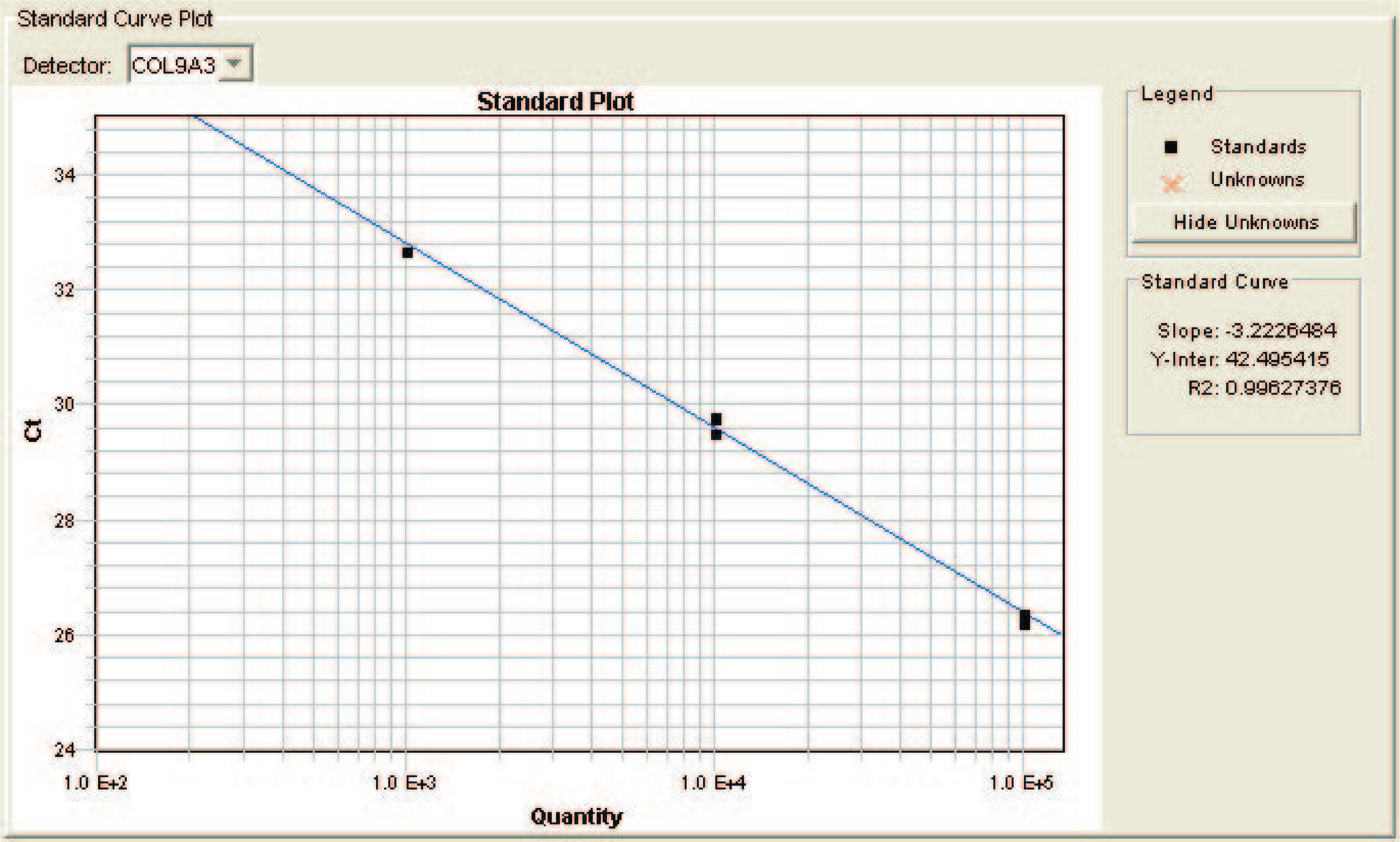
Standard Curve Plot
Detector: COL9A3
Standard Plot
Ct
34
32
30
28
26
24
1.0 E+2
1.0 E+3
1.0 E+4
1.0 E+5
Quantity
Legend
Standards
Unknowns
Hide Unknowns
Standard Curve
Slope: -3.2226484
Y-Inter: 42.495415
R2: 0.99627376

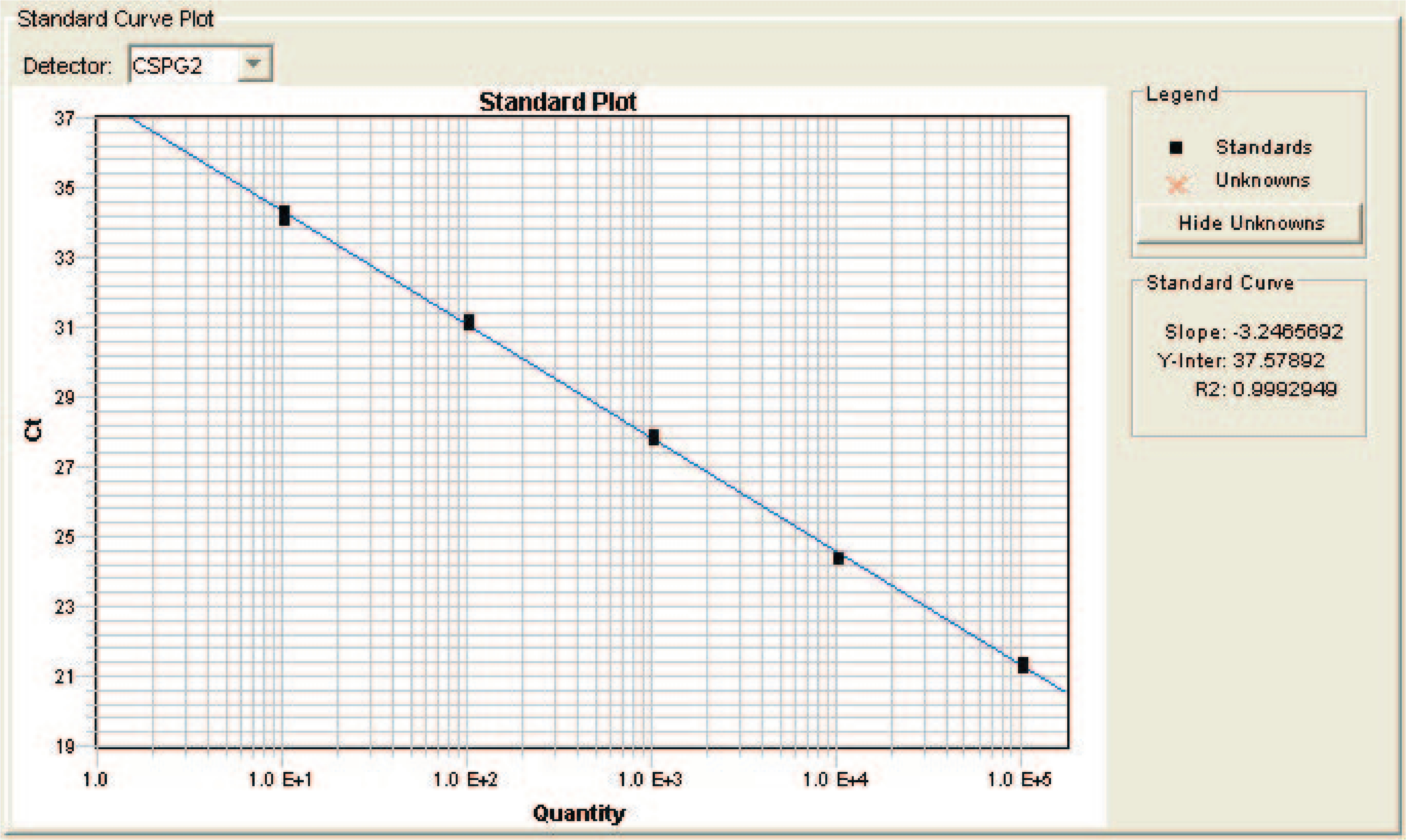
Standard Curve Plot
Detector: CSPG2
Standard Plot
Ct
37
35
33
31
29
27
25
23
21
19
1.0
1.0 E+1
1.0 E+2
1.0 E+3
1.0 E+4
1.0 E+5
Quantity
Legend
Standards
Unknowns
Hide Unknowns
Standard Curve
Slope: -3.2465692
Y-Inter: 37.57892
R2: 0.9992949

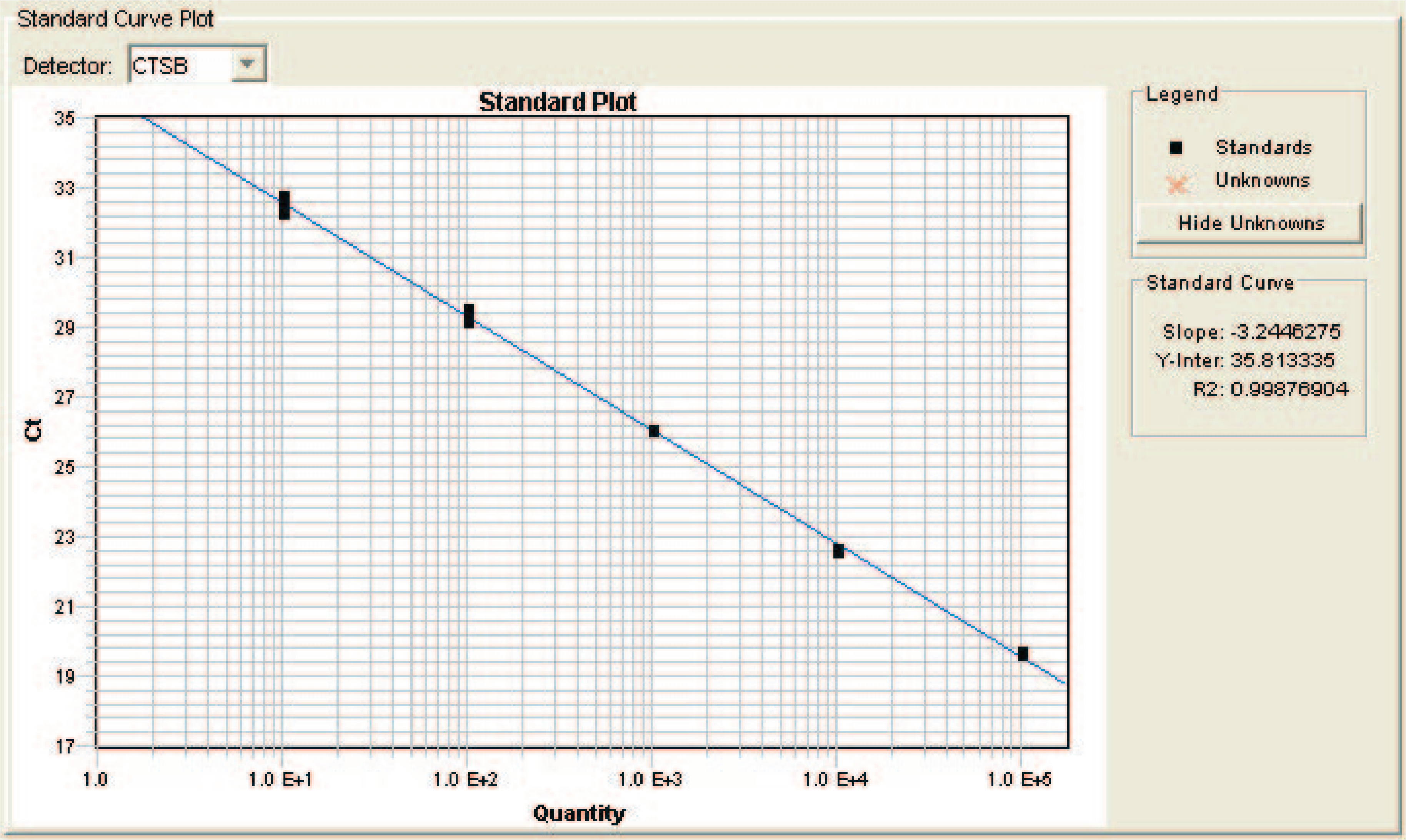

Standard Curve Plot
Detector: CTSB
Standard Plot
35
33
31
29
27
25
23
21
19
17
Ct
1.0
1.0 E+1
1.0 E+2
1.0 E+3
1.0 E+4
1.0 E+5
Quantity
Legend
Standards
Unknowns
Hide Unknowns
Standard Curve
Slope: -3.2446275
Y-Inter: 35.813335
R2: 0.99876904

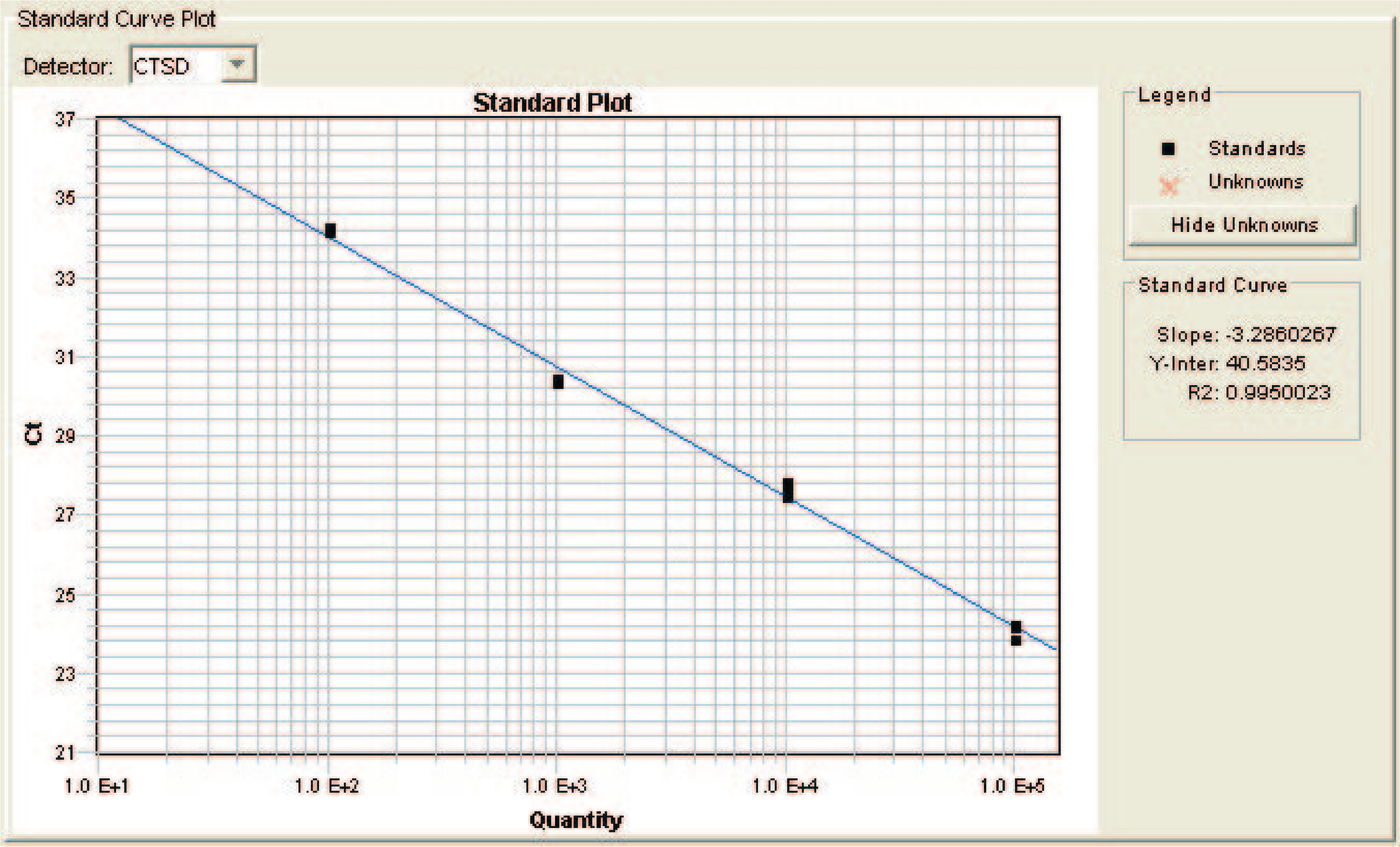
Standard Curve Plot
Detector: CTSD
Standard Plot
Ct
37
35
33
31
29
27
25
23
21
1.0 E+1
1.0 E+2
1.0 E+3
1.0 E+4
1.0 E+5
Quantity
Legend
Standards
Unknowns
Hide Unknowns
Standard Curve
Slope: -3.2860267
Y-Inter: 40.5835
R2: 0.9950023

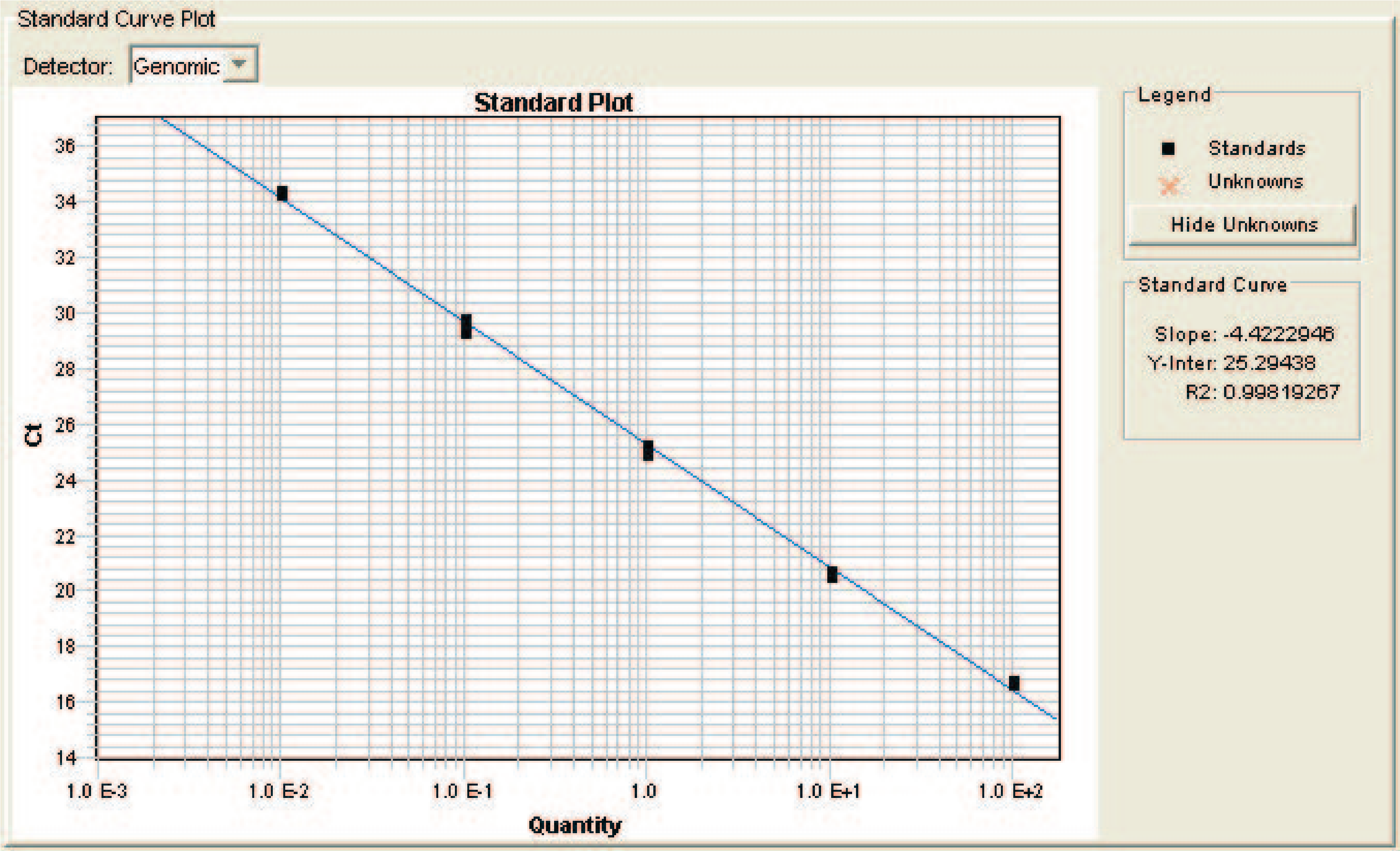
Standard Curve Plot
Detector: Genomic
Standard Plot
Ct
36
34
32
30
28
26
24
22
20
18
16
14
1.0 E-3
1.0 E-2
1.0 E-1
1.0
1.0 E+1
1.0 E+2
Quantity
Legend
Standards
Unknowns
Hide Unknowns
Standard Curve
Slope: -4.4222946
Y-Inter: 25.29438
R2: 0.99819267

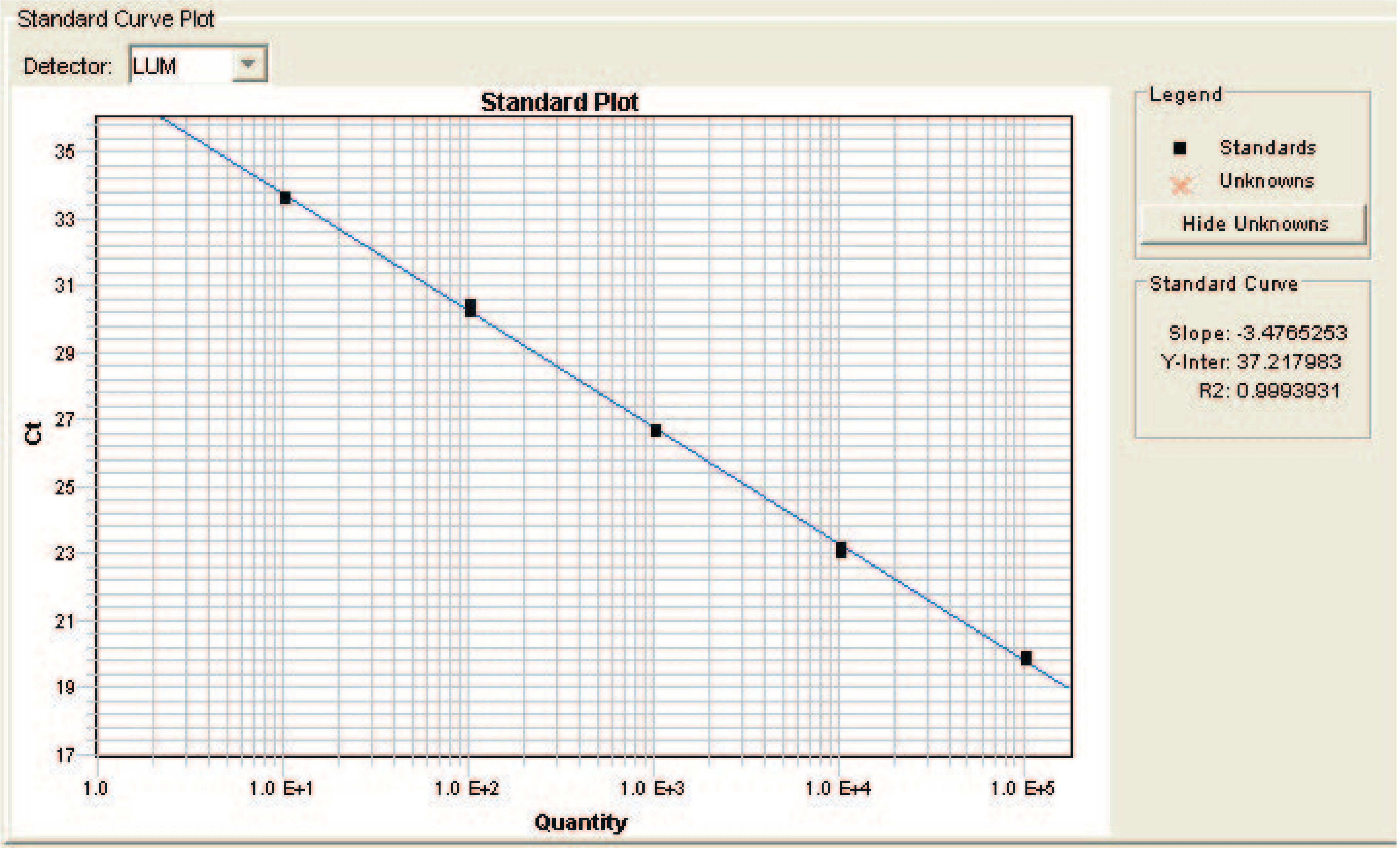

Standard Curve Plot
Detector: LUM
Standard Plot
Ct
35
33
31
29
27
25
23
21
19
17
1.0
1.0 E+1
1.0 E+2
1.0 E+3
1.0 E+4
1.0 E+5
Quantity
Legend
Standards
Unknowns
Hide Unknowns
Standard Curve
Slope: -3.4765253
Y-Inter: 37.217983
R2: 0.9993931

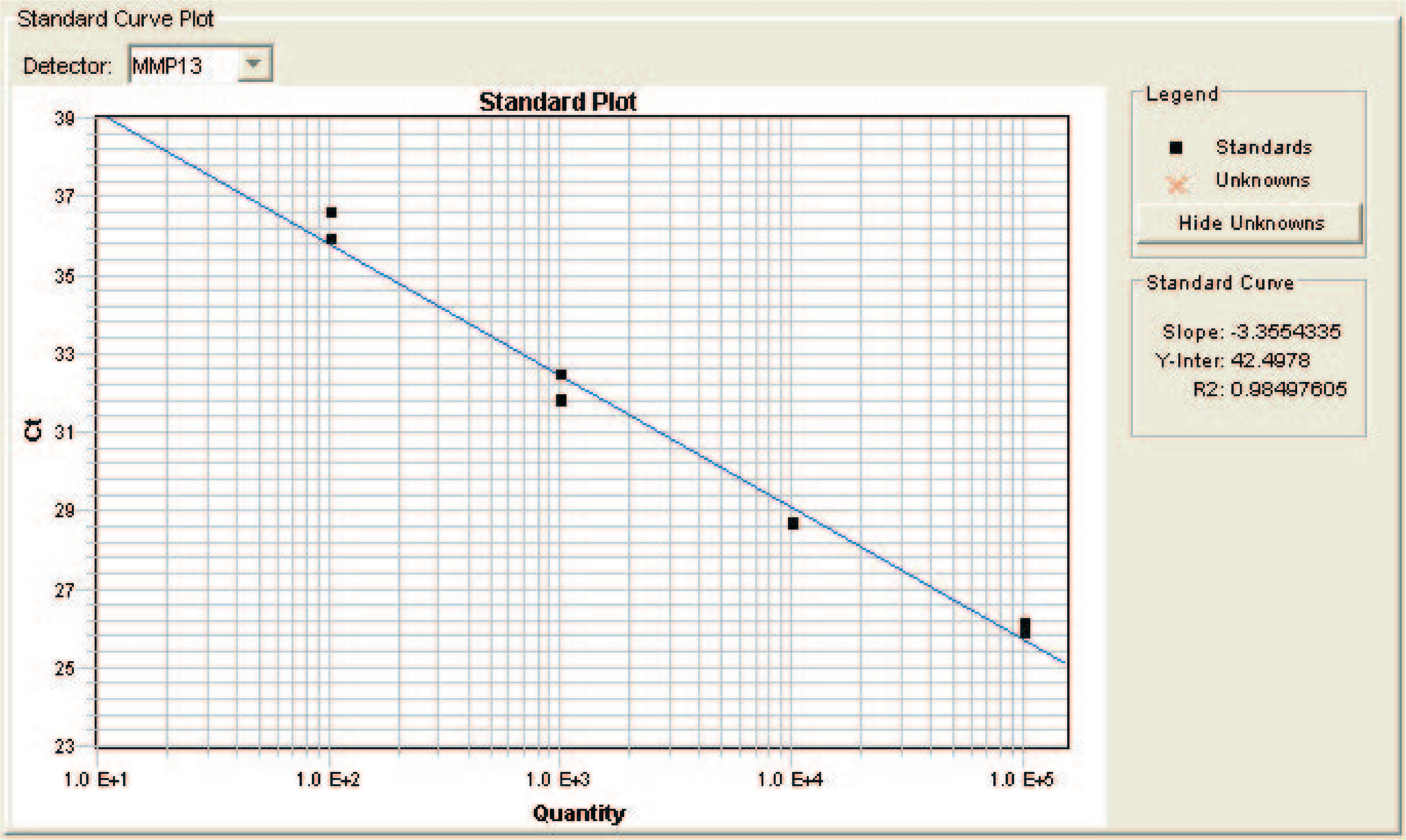
Standard Curve Plot
Detector: MMP13
Standard Plot
39
37
35
33
31
29
27
25
23
Ct
1.0 E+1
1.0 E+2
1.0 E+3
1.0 E+4
1.0 E+5
Quantity
Legend
Standards
Unknowns
Hide Unknowns
Standard Curve
Slope: -3.3554335
Y-Inter: 42.4978
R2: 0.98497605

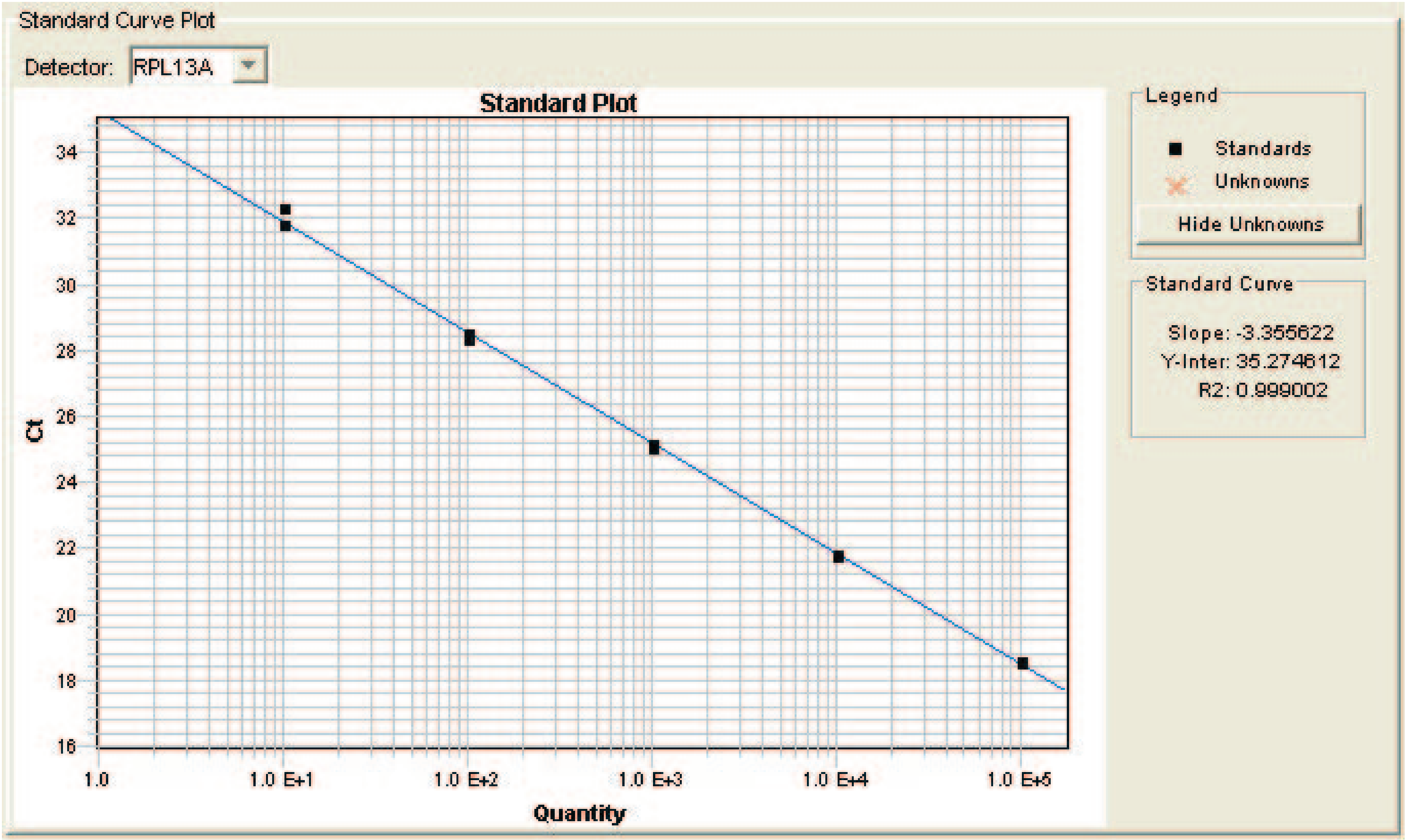
Standard Curve Plot
Detector: RPL13A
Standard Plot
Ct
34
32
30
28
26
24
22
20
18
16
1.0
1.0 E+1
1.0 E+2
1.0 E+3
1.0 E+4
1.0 E+5
Quantity
Legend
Standards
Unknowns
Hide Unknowns
Standard Curve
Slope: -3.355622
Y-Inter: 35.274612
R2: 0.999002

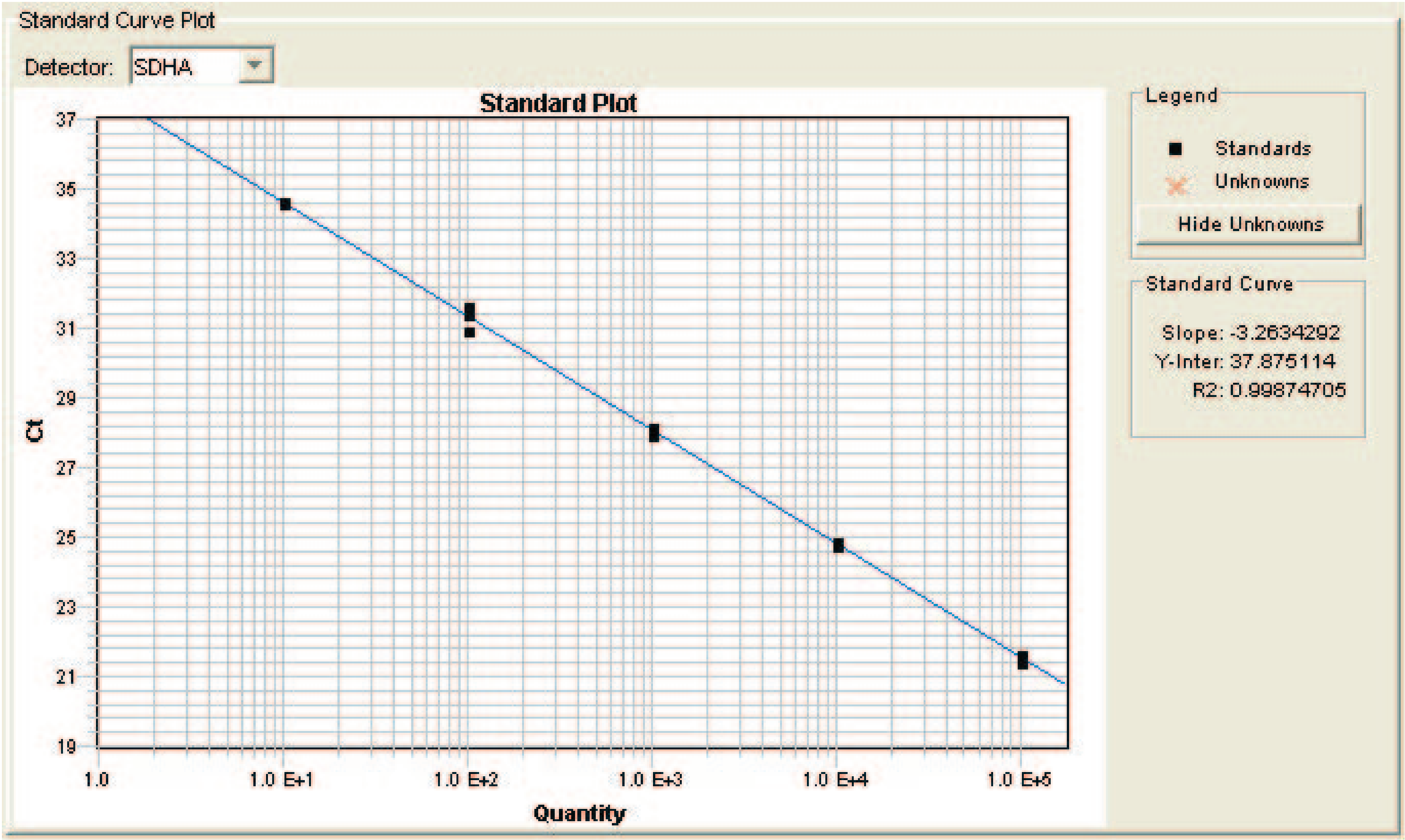
Standard Curve Plot
Detector: SDHA
Standard Plot
Ct
37
35
33
31
29
27
25
23
21
19
1.0
1.0 E+1
1.0 E+2
1.0 E+3
1.0 E+4
1.0 E+5
Quantity
Legend
Standards
Unknowns
Hide Unknowns
Standard Curve
Slope: -3.2634292
Y-Inter: 37.875114
R2: 0.99874705

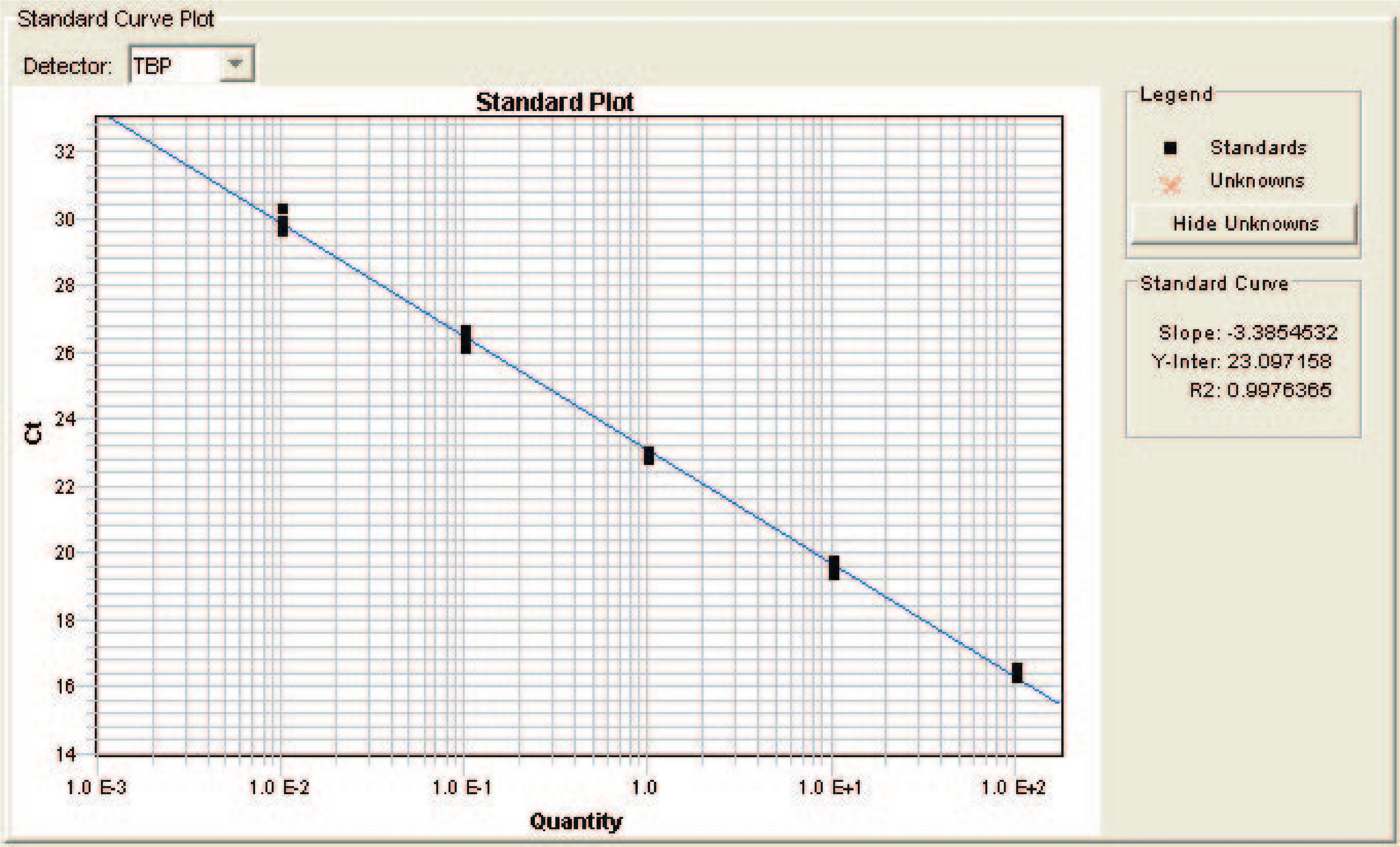
Standard Curve Plot
Detector: TBP
Standard Plot
Ct
32
30
28
26
24
22
20
18
16
14
1.0 E-3
1.0 E-2
1.0 E-1
1.0
1.0 E+1
1.0 E+2
Quantity
Legend
Standards
Unknowns
Hide Unknowns
Standard Curve
Slope: -3.3854532
Y-Inter: 23.097158
R2: 0.9976365

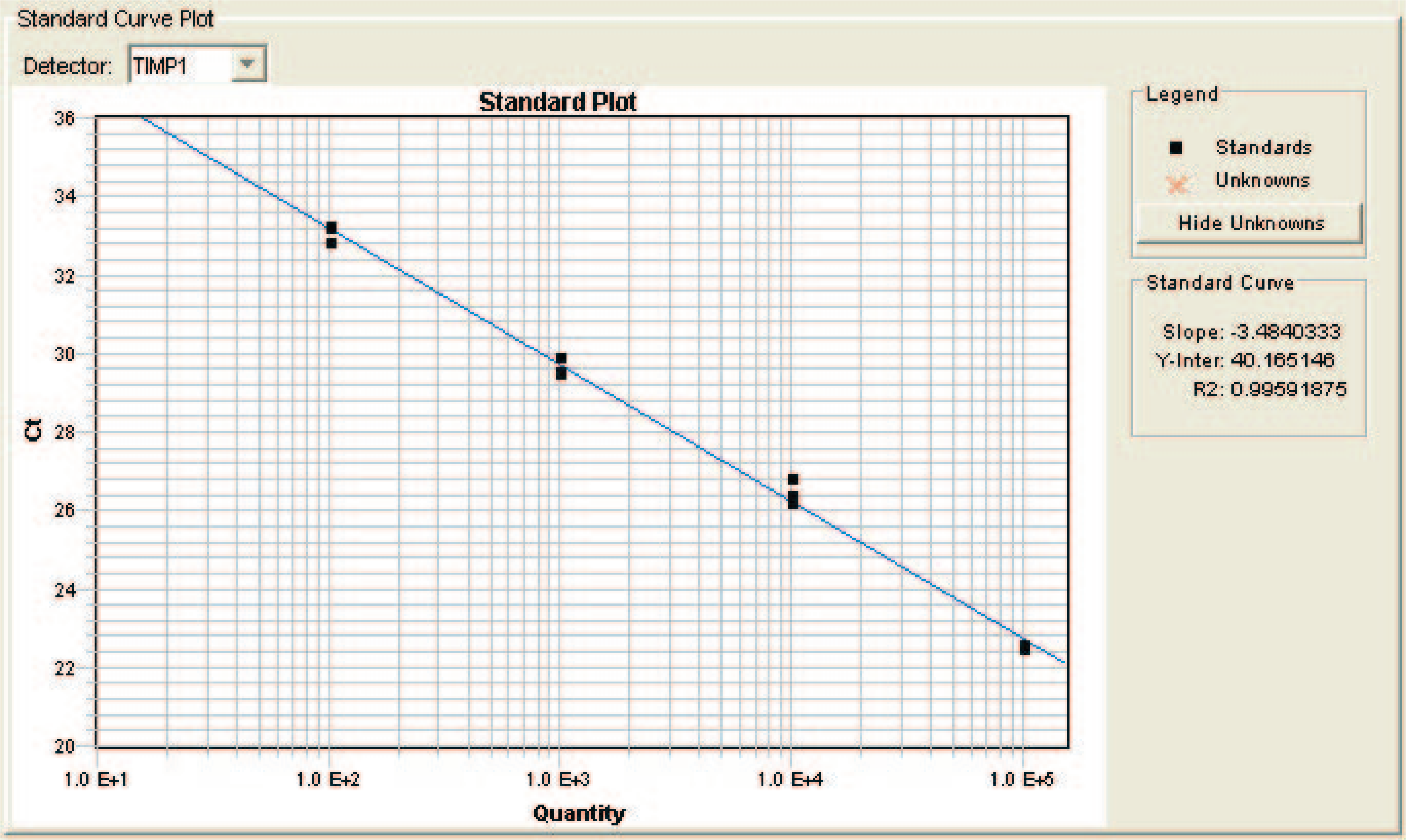
Standard Curve Plot
Detector: TIMP1
Standard Plot
Ct
36
34
32
30
28
26
24
22
20
1.0 E+1
1.0 E+2
1.0 E+3
1.0 E+4
1.0 E+5
Quantity
Legend
Standards
Unknowns
Hide Unknowns
Standard Curve
Slope: -3.4840333
Y-Inter: 40.165146
R2: 0.99591875

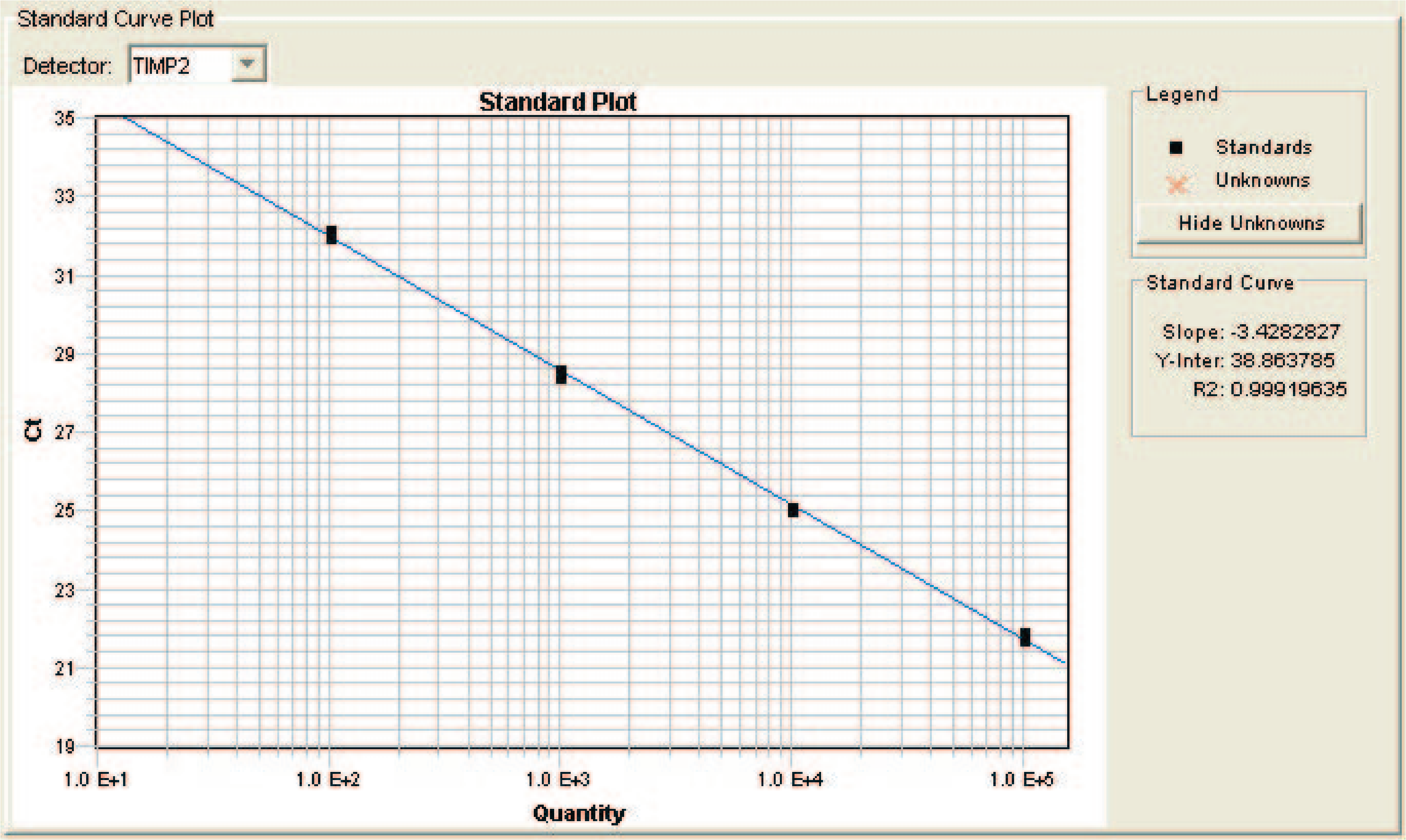

Standard Curve Plot
Detector: TIMP2
Standard Plot
Ct
35
33
31
29
27
25
23
21
19
1.0 E+1
1.0 E+2
1.0 E+3
1.0 E+4
1.0 E+5
Quantity
Legend
Standards
Unknowns
Hide Unknowns
Standard Curve
Slope: -3.4282827
Y-Inter: 38.863785
R2: 0.99919635

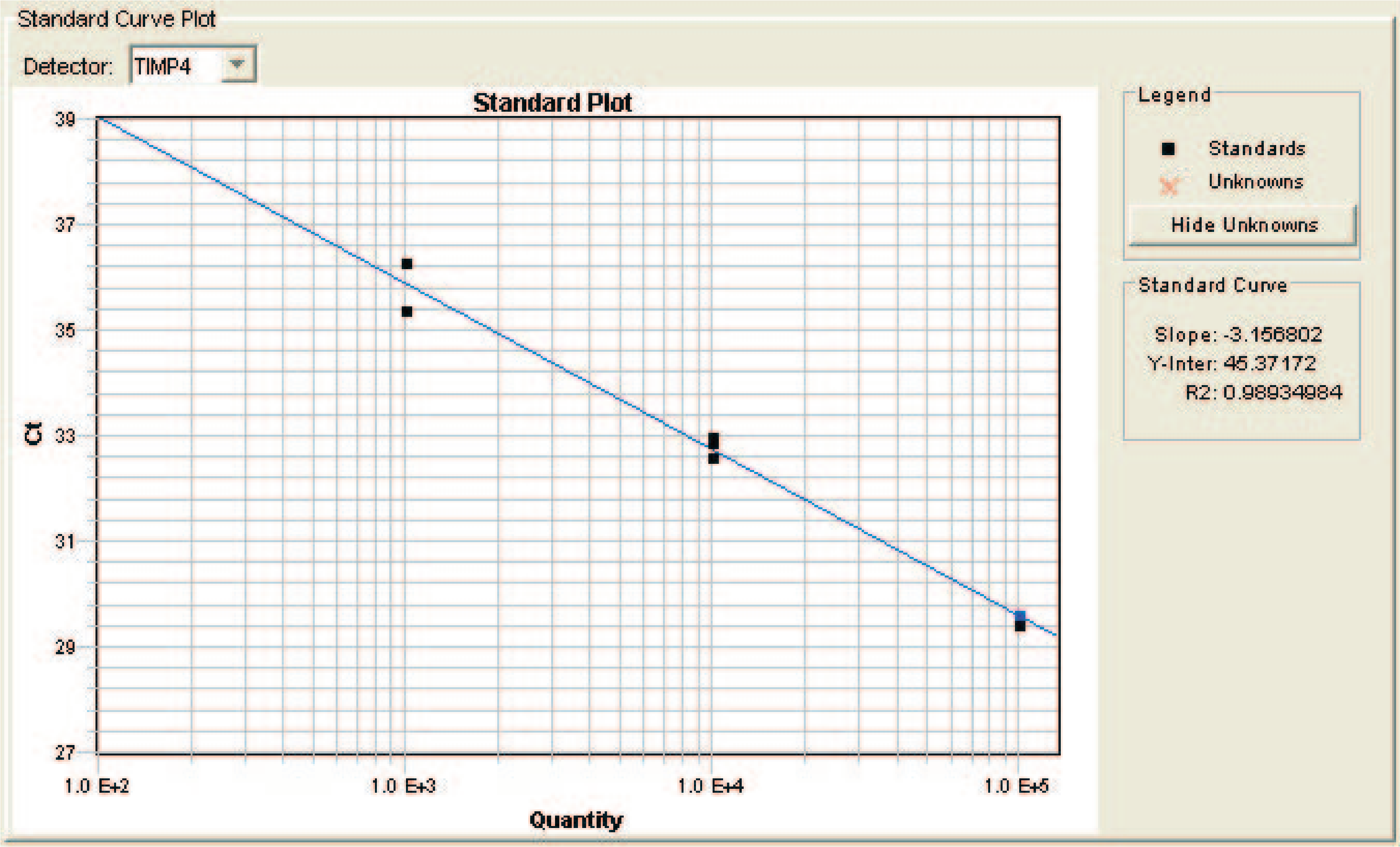
Standard Curve Plot
Detector: TIMP4
Standard Plot
39
37
35
33
31
29
27
Ct
1.0 E+2
1.0 E+3
1.0 E+4
1.0 E+5
Quantity
Legend
Standards
Unknowns
Hide Unknowns
Standard Curve
Slope: -3.156802
Y-Inter: 45.37172
R2: 0.98934984

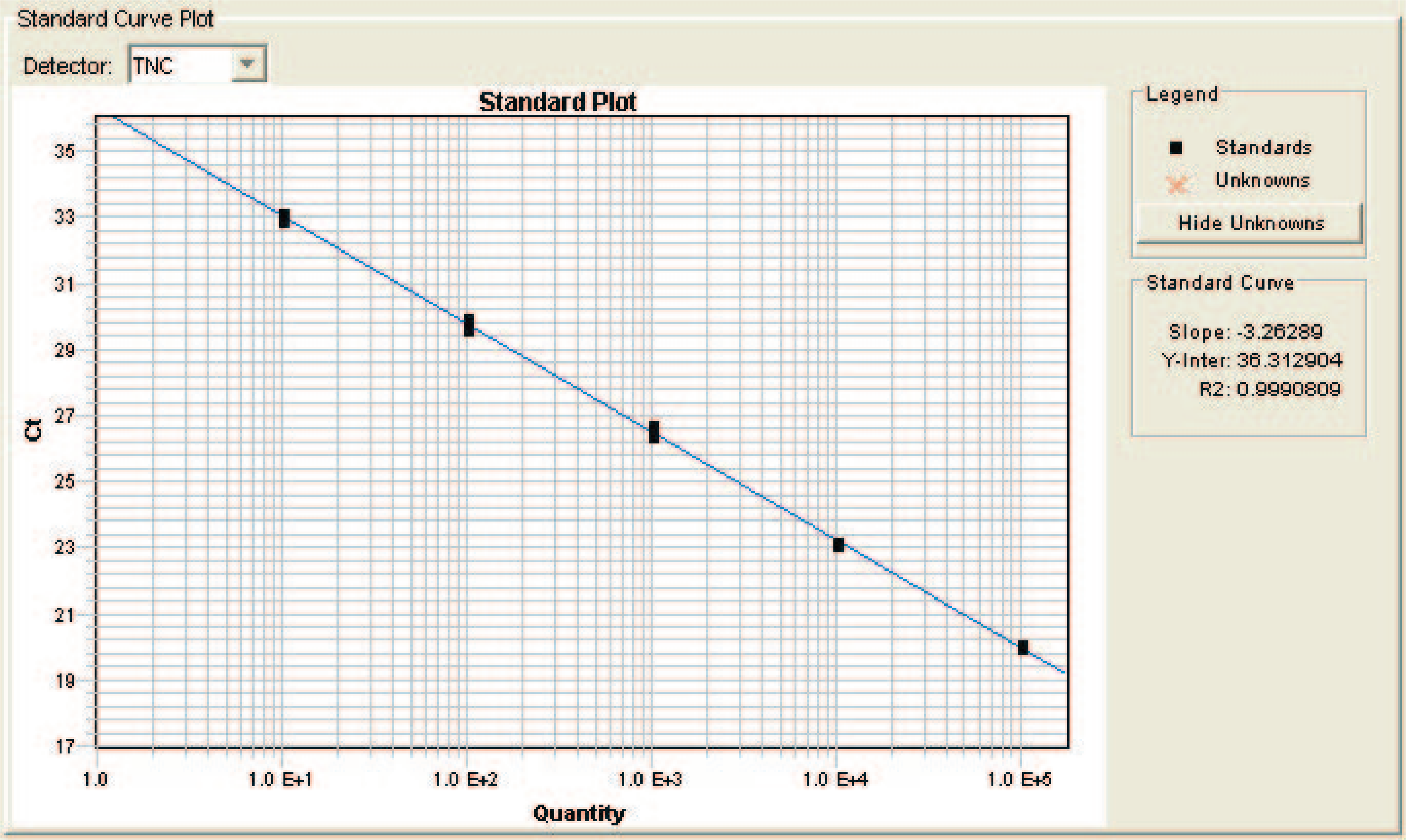
Standard Curve Plot
Detector: TNC
Standard Plot
Ct
35
33
31
29
27
25
23
21
19
17
1.0
1.0 E+1
1.0 E+2
1.0 E+3
1.0 E+4
1.0 E+5
Quantity
Legend
Standards
Unknowns
Hide Unknowns
Standard Curve
Slope: -3.26289
Y-Inter: 36.312904
R2: 0.9990809

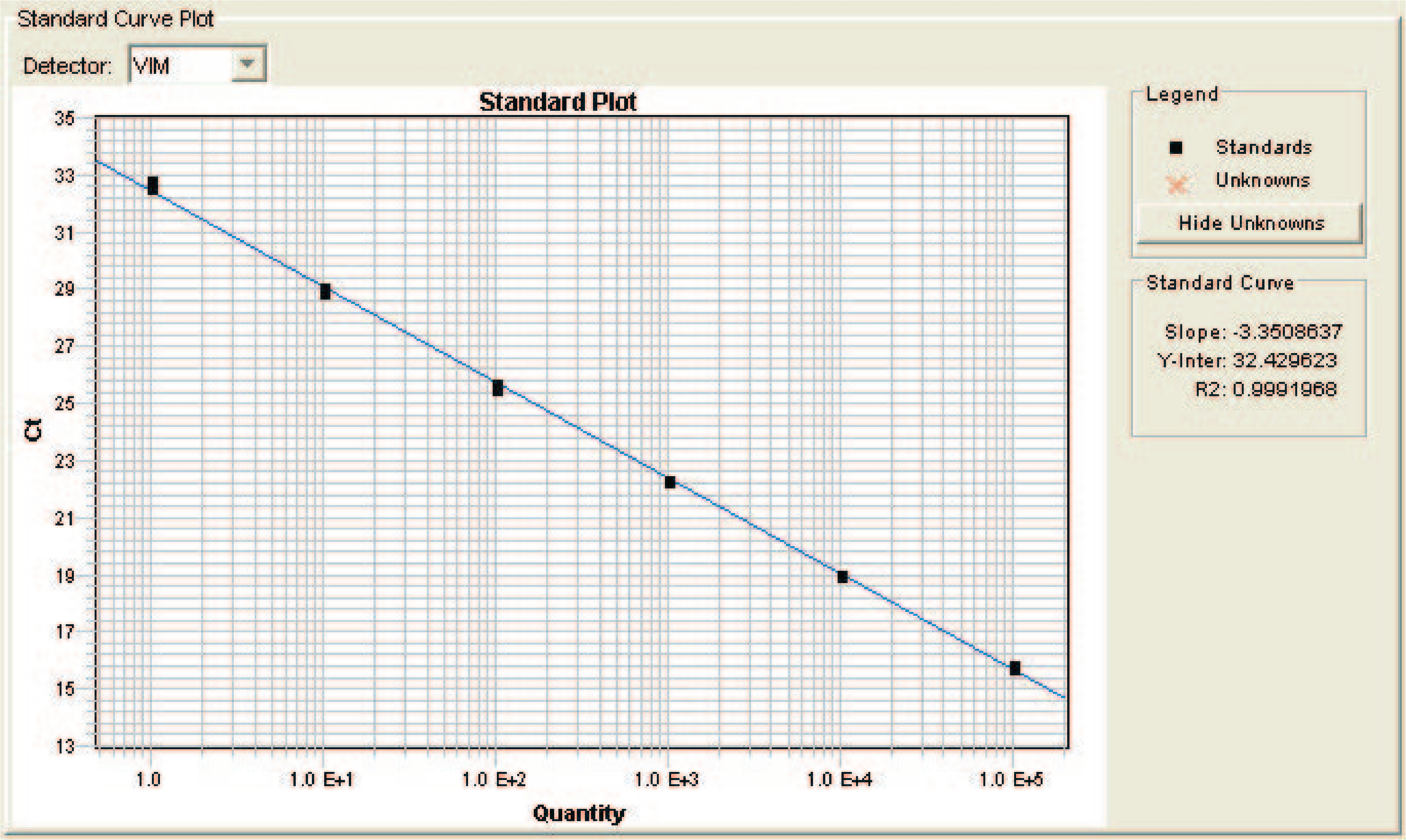
Standard Curve Plot
Detector: VIM
Standard Plot
Ct
35
33
31
29
27
25
23
21
19
17
15
13
1.0
1.0 E+1
1.0 E+2
1.0 E+3
1.0 E+4
1.0 E+5
Quantity
Legend
Standards
Unknowns
Hide Unknowns
Standard Curve
Slope: -3.3508637
Y-Inter: 32.429623
R2: 0.9991968

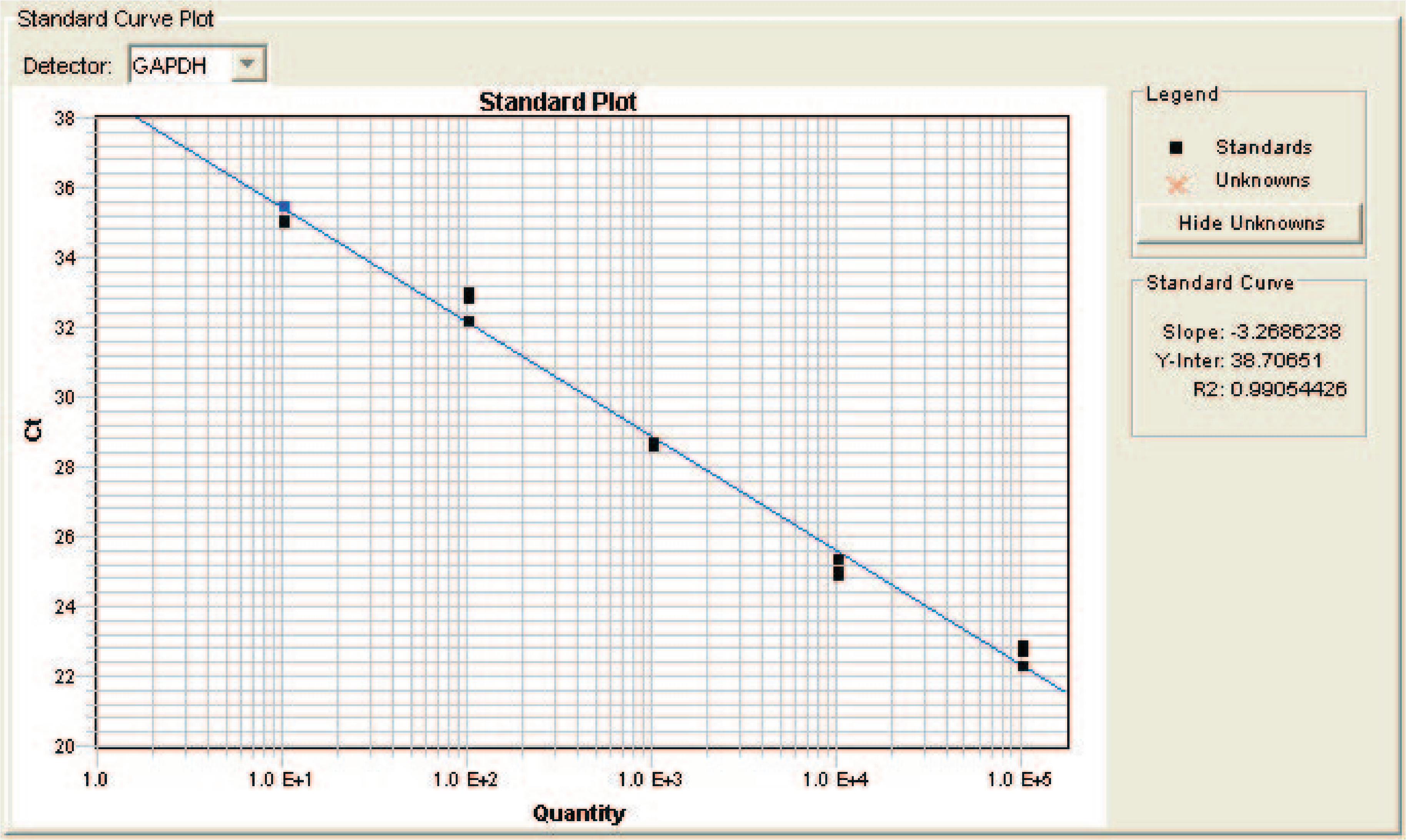
Standard Curve Plot
Detector: GAPDH
Standard Plot
Ct
38
36
34
32
30
28
26
24
22
20
1.0
1.0 E+1
1.0 E+2
1.0 E+3
1.0 E+4
1.0 E+5
Quantity
Legend
Standards
Unknowns
Hide Unknowns
Standard Curve
Slope: -3.2686238
Y-Inter: 38.70651
R2: 0.99054426

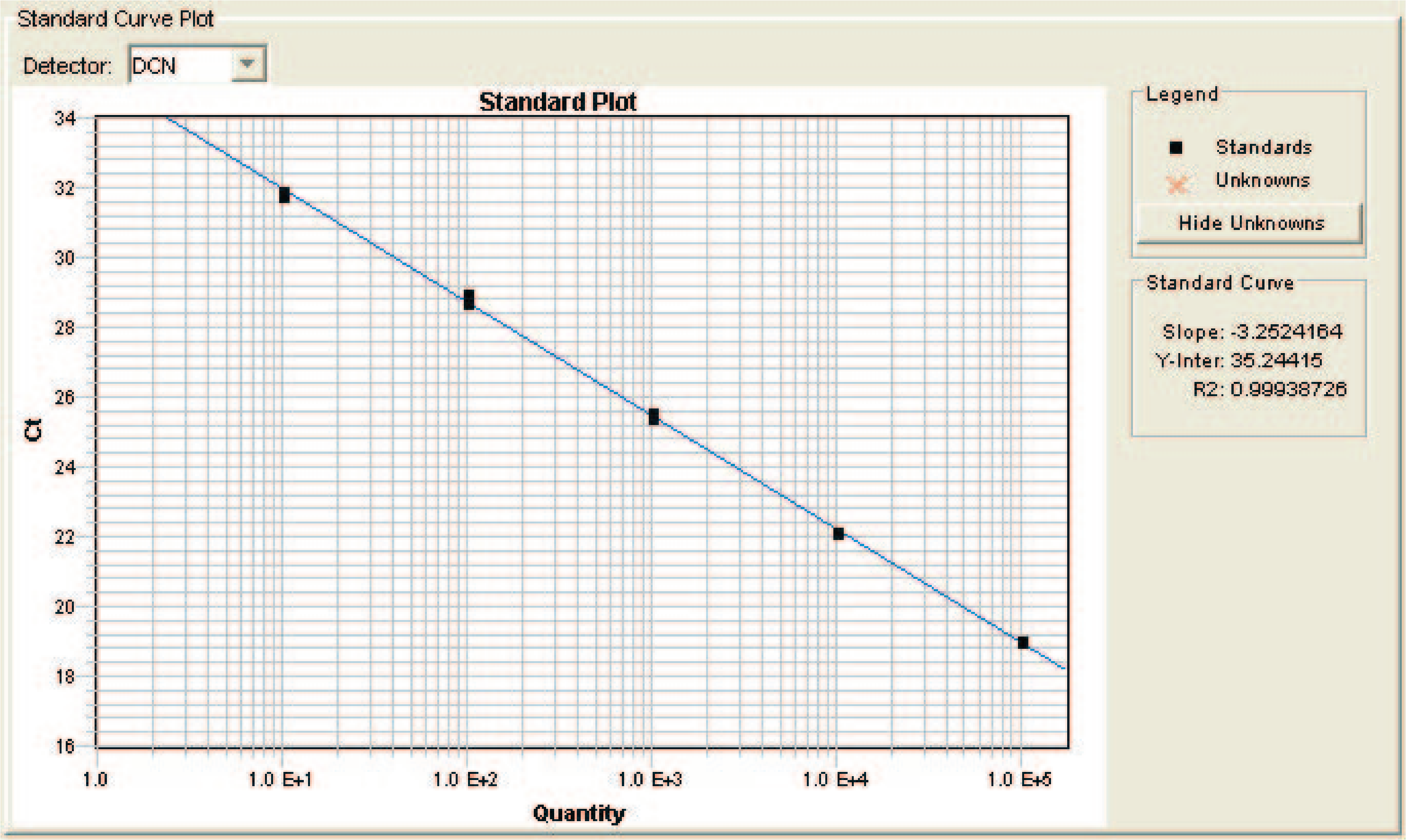

Standard Curve Plot
Detector: DCN
Standard Plot
Ct
34
32
30
28
26
24
22
20
18
16
1.0
1.0 E+1
1.0 E+2
1.0 E+3
1.0 E+4
1.0 E+5
Quantity
Legend
Standards
Unknowns
Hide Unknowns
Standard Curve
Slope: -3.2524164
Y-Inter: 35.24415
R2: 0.99938726
